# Supplementary material for: Formation of an Amyloid-like Structure During In Vitro Interaction of Titin and Myosin-Binding Protein C
Source: Int J Mol Sci. 2025 Jul 18;26(14):6910. doi: 10.3390/ijms26146910 (PMC12294915; doi:10.3390/ijms26146910)
Supplement: Supplementary file 1 [file ijms-26-06910-s001.zip › Supplementary Figures.pdf]

Suppl. Figure S1 Isolation of MyBP-C from rabbit skeletal muscle

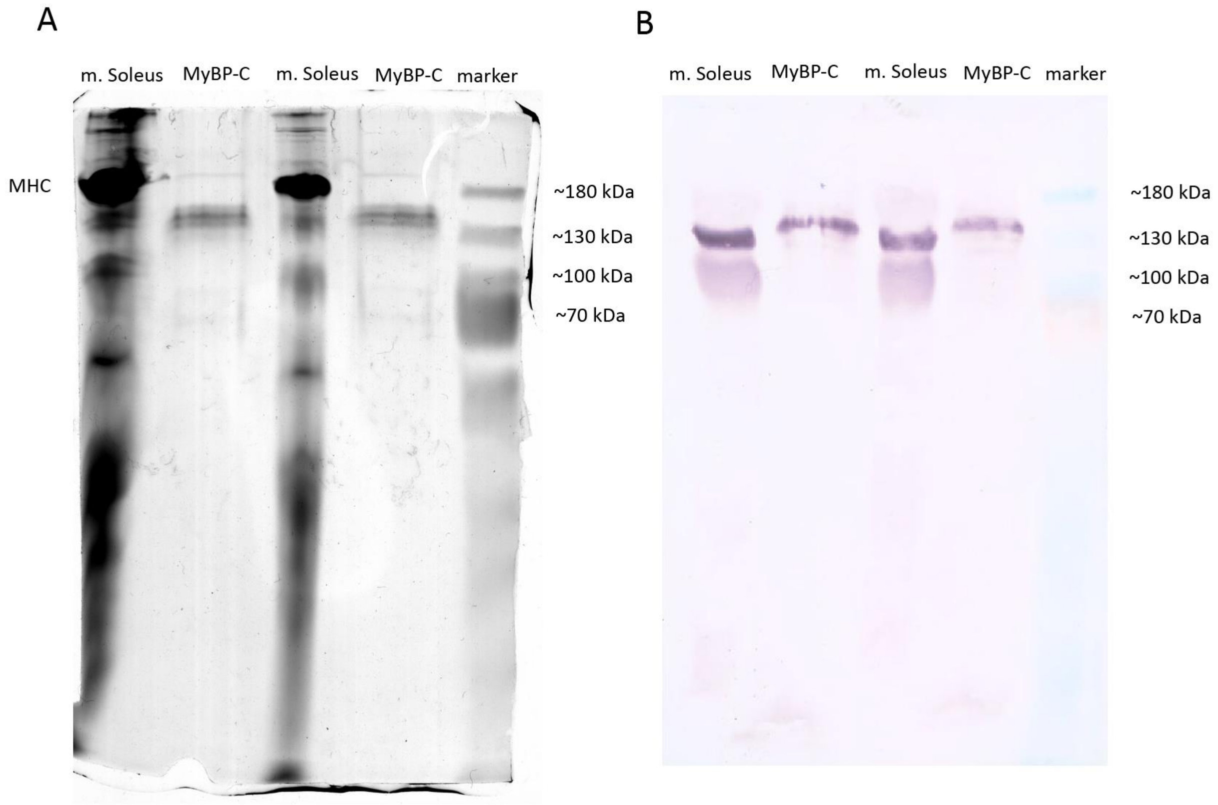

Figure S1. SDS-PAGE and Western blot analysis of myosin-binding MyBPC. A – SDS-PAGE of the purified MyBPC preparation (lanes 2 and 4). Lanes 1 and 3: rabbit soleus muscle extract (control). Based on electrophoretic mobility, the molecular weight of skeletal muscle MyBPC from rabbit is estimated between 125–134 kDa. Rightmost lane: protein molecular-weight markers. B – Western blot of C-protein using monoclonal anti-MYBPC2 antibodies. MHC – myosin heavy chains.

Preparations of MyBP-C were obtained. Electrophoretic analysis determined that the purity of the protein exceeded 80%.

In the gel of the purified MyBPC preparation, two protein bands are visible, which likely correspond to one of the paralogs of this protein and its degradation product (Figure 1A). Densitometric analysis indicates that the molecular weight of the upper band is approximately 134 kDa and constitutes about 82% of the total MyBPC. The lower band has a molecular weight of approximately 125 kDa and represents approximately 18% of the C-protein preparation.

Western blotting performed with monoclonal antibodies anti-MYBPC1 and anti-MYBPC2 identified the fast paralog of the MyBPC (Figure 1B).

Suppl. Figure S2 Isolation of titin from rabbit skeletal muscle

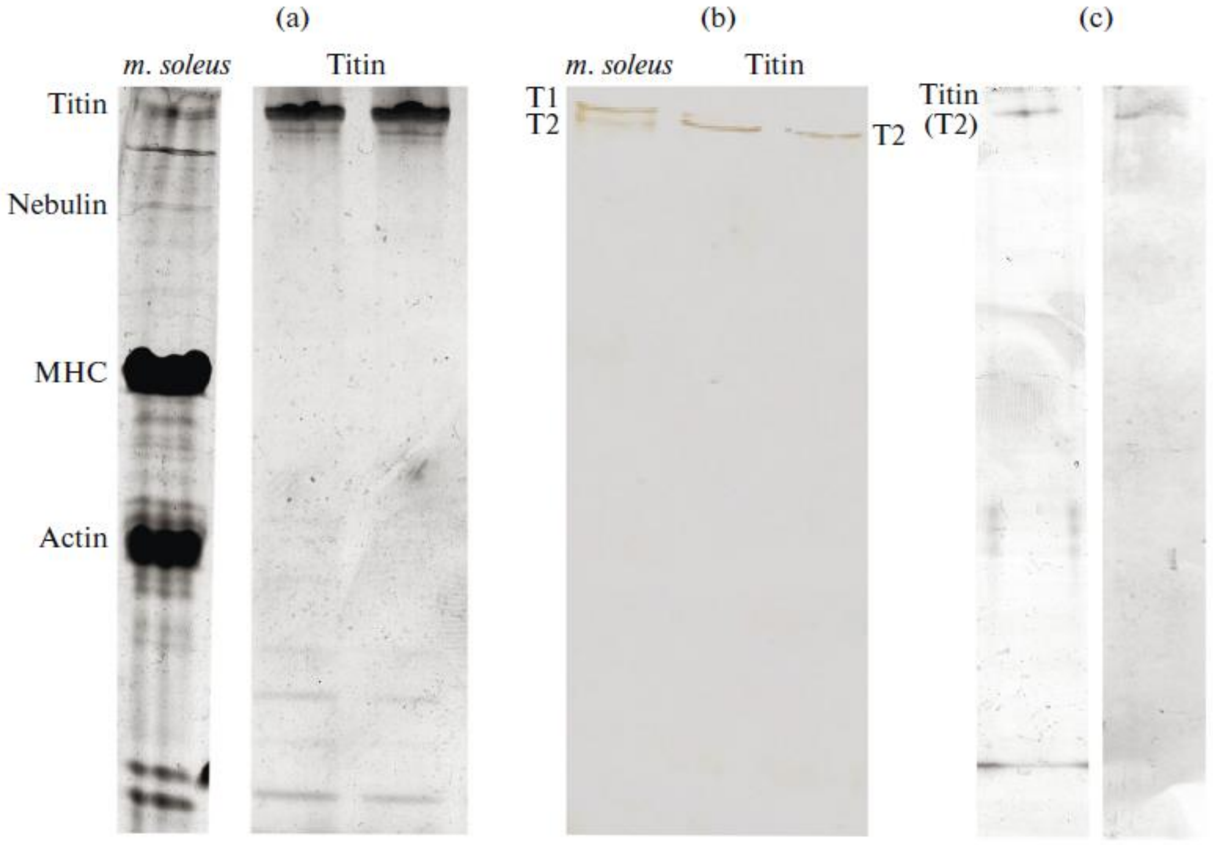

Figure S2. SDS–PAGE and Western blot analysis of rabbit skeletal muscle titin. A – SDS–PAGE of the purified titin preparation (two rightmost lanes) and rabbit soleus muscle extract (control, left lane). Gel electrophoresis was performed in a 7 % polyacrylamide gel as per (Fritz et al., 1989). Bands corresponding to actin, myosin heavy chains (MHC), nebulin, and titin are indicated. B – Western blot analysis of titin using monoclonal antibody AB5. Electrophoresis was carried out in a 2.2 % polyacrylamide gel reinforced with agarose. Left lane: rabbit soleus muscle extract (control); two right lanes: purified titin preparations. C – SDS–PAGE of purified titin (left lane) in a 7 % polyacrylamide gel, and Western blot detection of titin using monoclonal antibody 9D10 (right lane). T1: Full-length titin molecules spanning from the M-line to the Z-disk within the sarcomere. T2: Titin-1 fragments associated with the A-band region of the sarcomere along the myosin filaments.

On the gel of the purified skeletal titin preparation, a distinct band corresponding to titin was observed at an approximate molecular weight of ~2300 kDa (Figure 2A). We demonstrated that, in addition to titin itself, our samples contain approximately 10% low–molecular–weight impurity. Accordingly, the purity of the isolated protein is estimated at ~90%. Western blot data utilizing the monoclonal antibodies 9D10 (Figure 2C, right lane) and AB5 (Figure 2B) confirmed that the isolated protein is indeed titin.

Suppl. Figure S3 Electron Microscopy of Titin–MyBPC Aggregates

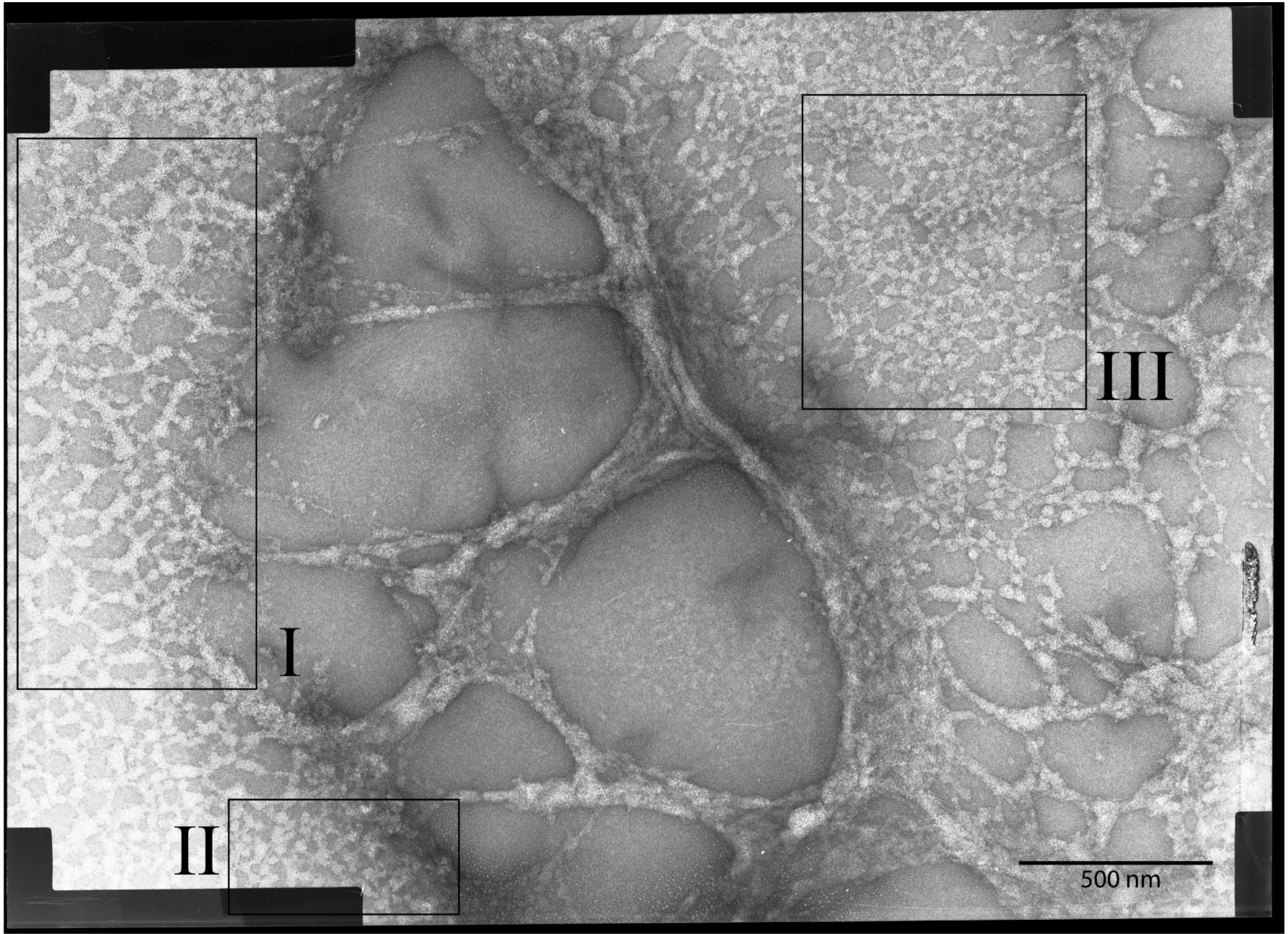

Suppl. Figure S4 Atomic Force Microscopy of Titin–MyBPC Aggregates

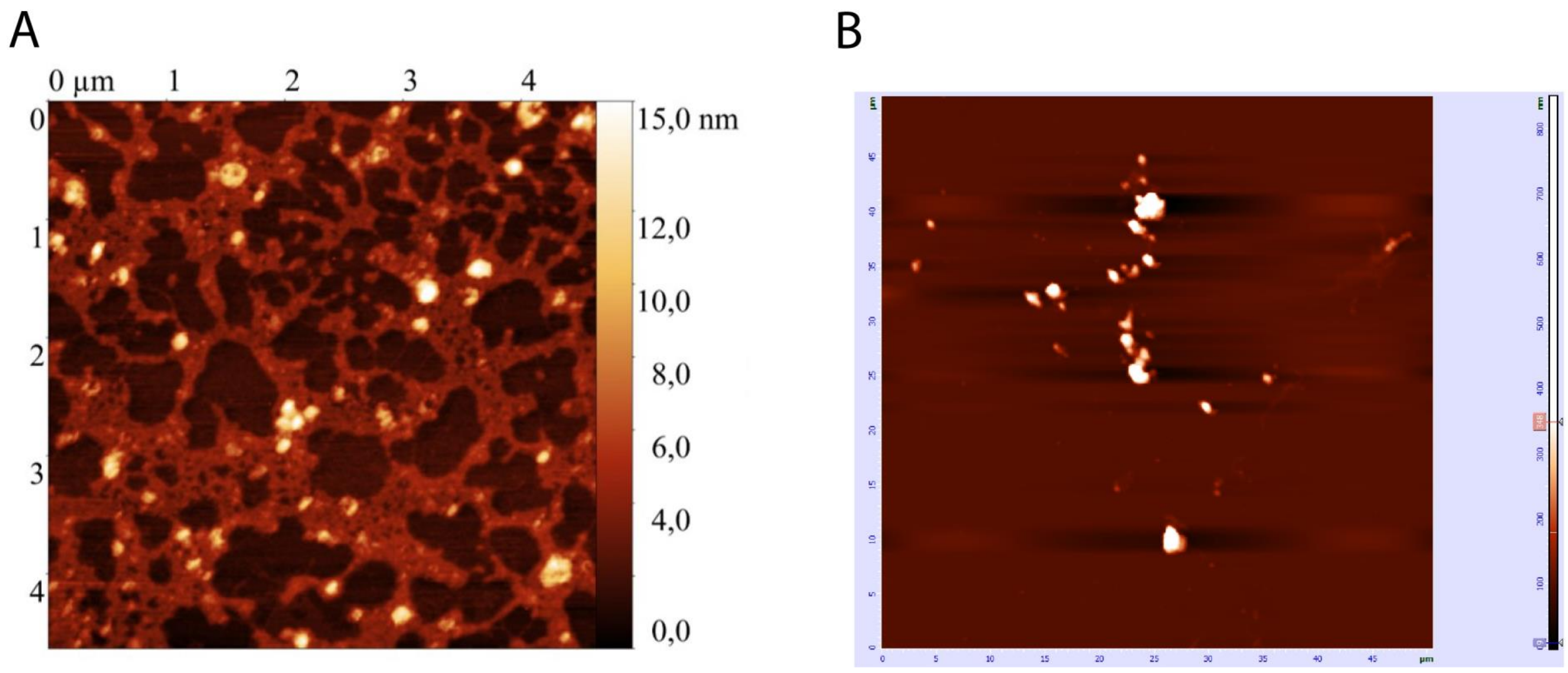

Figure 4. Aggregates of titin (A) and myosin-binding protein C (MyBPC) (B) were visualized by atomic force microscopy following incubation in a solution containing 200 mM KCl and 10 mM imidazole at pH 7.0. Both MyBPC and titin samples were dialyzed overnight for 24 h at 4°C at an initial concentration of 0.1 mg/mL.

Suppl. Figure S5. Atomic force microscopy of titin–MyBPC aggregates (analysis of images)

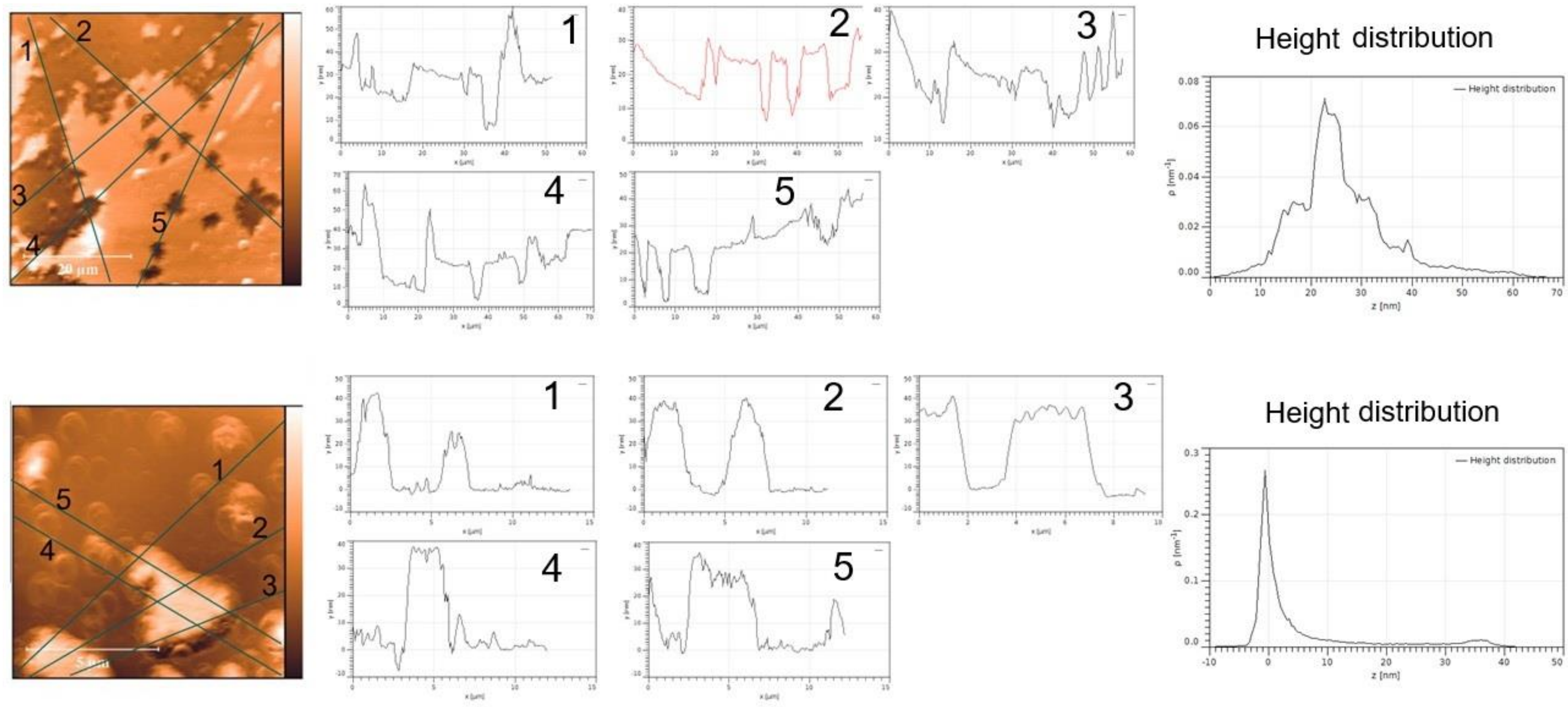

Suppl. Figure S6 Amino acid sequence identity

Titin (human)	FoldAmyloid	PASTA	AGGRESCAN	Waltz 2.0
**2, 63, Fn, 56**	0.11	0	0.09	0
**2, 64, Fn, 57**	0.16	0.17	0.07	0.06
**3, 65, Ig, 114**	0.28	0.42	0.34	0
**3, 74, Fn, 64**	0.24	0.23	0.13	0.07
**3, 75, Fn, 65**	0.19	0	0.33	0.06
**4, 76, Ig, 117**	0.23	0.41	0.44	0.07
**4, 85, Fn, 72**	0.28	0.16	0.27	0
**4, 86, Fn, 73**	0.19	0.14	0.38	0.12
**5, 87, Ig, 120**	0.2	0.32	0.36	0
**5, 96, Fn, 80**	0.29	0.06	0.26	0.16
**5, 97, Fn, 81**	0.26	0.09	0.27	0.05
**6, 98, Ig, 123**	0	0	0.22	0.12
**6, 107, Fn, 88**	0.34	0.05	0.25	0
**6, 108, Fn, 89**	0.07	0	0.24	0.06
**7, 109, Ig, 126**	0.27	0.43	0.43	0
**7, 118, Fn, 96**	0.22	0	0.19	0
**7, 119, Fn, 97**	0.14	0.16	0.32	0
**8, 120, Ig, 129**	0.17	0	0.32	0
**8, 129, Fn, 104**	0.23	0.15	0.29	0
**8, 130, Fn, 105**	0.12	0.19	0.36	0.07
**9, 131, Ig, 131**	0.05	0.11	0.22	0.12
**9, 140, Fn, 112**	0.27	0.23	0.19	0
**9, 141, Fn, 113**	0.14	0.06	0.34	0.08
**10, 142, Ig, 134**	0.08	0	0.09	0.07
**10, 151, Fn, 120**	0.24	0.07	0.34	0.05
**10, 152, Fn, 121**	0.23	0.06	0.26	0
**11, 153, Ig, 137**	0.19	0.07	0.37	0.07

MyBP-C cardiac (human)	FoldAmyloid	PASTA	AGGRESCAN	Waltz 2.0
**8, Ig, 6**	0.23	0.19	0.26	0
**9, Fn, 3**	0.35	0.2	0.43	0.18
**10, Ig, 7**	0.28	0.19	0.38	0
MyBP-C fast skeletal (human)				
**8, Ig, 6**	0.24	0.29	0.29	0.06
**9, Fn, 3**	0.25	0.16	0.38	0.24
**10, Ig, 7**	0.28	0.4	0.39	0

The average identity in the amino acid sequence between neighboring human titin FnIII domains and Ig domains and cardiac MyBP-C

MyBP-C cardiac (human) and titin (human)

8, Ig, 6

PRLQLPRHLRQTIQKKVGEPVNLLIPFQGKPRPQVTWTKEGQPLAGEEVSIRNSPTDTILFIRAARRVHSGTYQVTVRIENMEDKATLVLQVVDK

3, 65, Ig, 114

PDFELDAELRRTLVVRAGLSIRIFVPIKGRPAPEVTWTKDNINLKNRANIENTESFTLLIIPECNRYDTGKFVMTIENPAGKKSGFVNVRVLD

Score	Expect	Method	Identities	Positives	Gaps
51.6 bits(122)	3e-15	Compositional matrix adjust.	29/92(32%)	49/92(53%)	3/92(3%)

```
Query  1   PRLQLPRHLRQTIQKKVGEPVNLLIPFQGKPRPQVTWTKEGQPLAGEEVSIRNSPTDTIL  60
            P  +L   LR+T+  + G  + + +P +G+P P+VTWTK+   L     +I N+ + T+L
Sbjct  1   PDFELDAELRRTLVVRAGLSIRIFVPIKGRPAPEVTWTKDNINLKN-RANIENTESFTLL  59

Query  61  FIRAARRVHSGTYQVTVRIENMEDKATLVLQV  92
             I    R  +G + +T  IEN   K +  + V
Sbjct  60  IIPECNRYDTGKFVMT--IENPAGKKSGFVNV  89
```

8, Ig, 6

PRLQLPRHLRQTIQKKVGEPVNLLIPFQGKPRPQVTWTKEGQPLAGEEVSIRNSPTDTILFIRAARRVHSGTYQVTVRIENMEDKATLVLQVVDK

4, 76, Ig, 117

PEIELDADLRKVVTIRACCTLRLFVPIKGRPAPEVKWARDHGESLDKASIESTSSYTLLIVGNVNRFDSGKYILTVENSSGSKSAFVNVR

Score	Expect	Method	Identities	Positives	Gaps
47.8 bits(112)	9e-14	Compositional matrix adjust.	26/93(28%)	49/93(52%)	5/93(5%)

```
Query  1   PRLQLPRHLRQTIQKKVGEPVNLLIPFQGKPRPQVTWTKE-GQPLAGEEVSIRNSPTDTI  59
           P ++L   LR+ +  +    + L +P +G+P P+V W ++ G+ L  ++ SI ++ + T+
Sbjct  1   PEIELDADLRKVVTIRACCTLRLFVPIKGRPAPEVKWARDHGESL--DKASIESTSSYTL  58

Query  60  LFIRAARRVHSGTYQVTVRIENMEDKATLVLQV  92
           L +    R  SG Y +TV  EN     +  + V
Sbjct  59  LIVGNVNRFDSGKYILTV--ENSSGSKSAFVNV  89
```

MyBP-C cardiac (human) and titin (human)

8, Ig, 6

PRLQLPRHLRQTIQKKVGEPVNLLIPFQGKPRPQVTWTKEGQPLAGEEVSIRNSPTDTILFIRAARRVHSGTYQVTVRIENMEDKATLVLQVVDK

5, 87, Ig, 120

PDIDLDLELRKIINIRAGGSLRLFVPIKGRPTPEVKWGKVDGEIRDAAIIDVTSSFTSLVLDNVNRYDSGKYTLTLENSSGTKSAFVT

Score	Expect	Method	Identities	Positives	Gaps
42.4 bits(98)	1e-11	Compositional matrix adjust.	23/78(29%)	39/78(50%)	3/78(3%)

```
Query  1    PRLQLPRHLRQTIQKKVGEPVNLLIPFQGKPRPQVTWTK-EGQPLAGEEVSIRNSPTDTI  59
            P + L   LR+ I  + G  + L +P +G+P P+V W K +G+      + + +S T  +
Sbjct  1    PDIDLDLELRKIINIRAGGSLRLFVPIKGRPTPEVKWGKVDGEIRDAAIIDVTSSFTSLV  60

Query  60   LFIRAARRVHSGTYQVTV  77
            L      R  SG Y +T+
Sbjct  61   L--DNVNRYDSGKYTLTL  76
```

8, Ig, 6

PRLQLPRHLRQTIQKKVGEPVNLLIPFQGKPRPQVTWTKEGQPLAGEEVSIRNSPTDTILFIRAARRVHSGTYQVTVRIENMEDKATLVLQVVDK

6, 98, Ig, 123

PELDLDSELRKGIVVRAGGSARIHIPFKGRPTPEITWSREEGEFTDKVQIEKGVNYTQLSIDNCDRNDAGKYILKLENSSGSKSAFVTVK

Score	Expect	Method	Identities	Positives	Gaps
45.8 bits(107)	4e-13	Compositional matrix adjust.	27/92(29%)	44/92(47%)	3/92(3%)

```
Query  1    PRLQLPRHLRQTIQKKVGEPVNLLIPFQGKPRPQVTWTKEGQPLAGEEVSIRNSPTDTIL  60
            P L L   LR+ I  + G    + IPF+G+P P++TW++E      ++V I      T L
Sbjct  1    PELDLDSELRKGIVVRAGGSARIHIPFKGRPTPEITWSREEGEFT-DKVQIEKGVNYTQL  59

Query  61   FIRAARRVHSGTYQVTVRIENMEDKATLVLQV  92
             I    R  +G Y   +++EN     +  + V
Sbjct  60   SIDNCDRNDAGKY--ILKLENSSGSKSAFVTV  89
```

MyBP-C cardiac (human) and titin (human)

8, Ig, 6

PRLQLPRHLRQTIQKKVGEPVNLLIPFQGKPRPQVTWTKEGQPLAGEEVSIRNSPTDTILFIRAARRVHSGTYQVTVRIENMEDKATLVLQVVDK

7, 109, Ig, 126

PEIELDADLRKVVVLRASATLRLFVTIKGRPEPEVKWEKAEGILTDRAQIEVTSSFTMLVIDNVTRFDSGRYNLTLENNSGSKTAFVNVR

Score	Expect	Method	Identities	Positives	Gaps
41.6 bits(96)	2e-11	Compositional matrix adjust.	24/92(26%)	43/92(46%)	3/92(3%)

```
Query  1    PRLQLPRHLRQTIQKKVGEPVNLLIPFQGKPRPQVTWTKEGQPLAGEEVSIRNSPTDTIL  60
            P ++L   LR+ +  +    + L +  +G+P P+V W K  + +  +   I  + + T+L
Sbjct  1    PEIELDADLRKVVVLRASATLRLFVTIKGRPEPEVKWEK-AEGILTDRAQIEVTSSFTML  59

Query  61   FIRAARRVHSGTYQVTVRIENMEDKATLVLQV  92
             I    R  SG Y +T  +EN     T  + V
Sbjct  60   VIDNVTRFDSGRYNLT--LENNSGSKTAFVNV  89
```

8, Ig, 6

PRLQLPRHLRQTIQKKVGEPVNLLIPFQGKPRPQVTWTKEGQPLAGEEVSIRNSPTDTILFIRAARRVHSGTYQVTVRIENMEDKATLVLQVVDK

8, 120, Ig, 129

PQIAKEREEEPLFDIDSEMRKTLIVKAGASFTMTVPFRGRPVPNVLWSKPDTDLRTRAYVDTTDSRTSLTIENANRNDSGKYTLTIQNVLSAASLT

Score	Expect	Method	Identities	Positives	Gaps
44.7 bits(104)	2e-12	Compositional matrix adjust.	27/89(30%)	43/89(48%)	3/89(3%)

```
Query  1    PRLQLPRHLRQTIQKKVGEPVNLLIPFQGKPRPQVTWTKEGQPLAGEEVSIRNSPTDTIL  60
            P   +   +R+T+  K G    + +PF+G+P P V W+K    L      +  + + T L
Sbjct  11   PLFDIDSEMRKTLIVKAGASFTMTVPFRGRPVPNVLWSKPDTDLRTRAY-VDTTDSRTSL  69

Query  61   FIRAARRVHSGTYQVTVRIENMEDKATLV  89
             I  A R  SG Y +T  I+N+   A+L
Sbjct  70   TIENANRNDSGKYTLT--IQNVLSAASLT  96
```

MyBP-C cardiac (human) and titin (human)

8, Ig, 6

PRLQLPRHLRQTIQKKVGEPVNLLIPFQGKPRPQVTWTKEGQPLAGEEVSIRNSPTDTILFIRAARRVHSGTYQVTVRIENMEDKATLVLQVVDK

8, 130, Fn, 105

PPAKIRIADSTKSSITLGWSKPVYDGGSAVTGYVVEIRQGEEEEWTTVSTKGEVRTTEYVVSNLKPGVNYYFRVSAVNCAGQGEPIEMNEPVQAKDI

Score	Expect	Method	Identities	Positives	Gaps
16.9 bits(32)	0.034	Compositional matrix adjust.	6/13(46%)	8/13(61%)	0/13(0%)

```
Query  18   GEPVNLLIPFQGK  30
            GEP+ +  P Q K
Sbjct  83   GEPIEMNEPVQAK  95
```

8, Ig, 6

PRLQLPRHLRQTIQKKVGEPVNLLIPFQGKPRPQVTWTKEGQPLAGEEVSIRNSPTDTILFIRAARRVHSGTYQVTVRIENMEDKATLVLQVVDK

9, 131, Ig, 131

PEIDLDVALRTSVIAKAGEDVQVLIPFKGRPPPTVTWRKDEKNLGSDARYSIENTDSSSLLTIPQVTRNDTGKYILTIENGVGEPKSSTVS

Score	Expect	Method	Identities	Positives	Gaps
56.2 bits(134)	5e-17	Compositional matrix adjust.	32/90(36%)	50/90(55%)	1/90(1%)

```
Query  1    PRLQLPRHLRQTIQKKVGEPVNLLIPFQGKPRPQVTWTKEGQPLAGEE-VSIRNSPTDTI  59
            P + L   LR ++  K GE V +LIPF+G+P P VTW K+ + L  +   SI N+ + ++
Sbjct  1    PEIDLDVALRTSVIAKAGEDVQVLIPFKGRPPPTVTWRKDEKNLGSDARYSIENTDSSSL  60

Query  60   LFIRAARRVHSGTYQVTVRIENMEDKATLV  89
            L I    R  +G Y +T+     E K++ V
Sbjct  61   LTIPQVTRNDTGKYILTIENGVGEPKSSTV  90
```

MyBP-C cardiac (human) and titin (human)

8, Ig, 6

PRLQLPRHLRQTIQKKVGEPVNLLIPFQGKPRPQVTWTKEGQPLAGEEVSIRNSPTDTILFIRAARRVHSGTYQVTVRIENMEDKATLVLQVVDK

10, 142, Ig, 134

PELDIDANFKQTHVVRAGASIRLFIAYQGRPTPTAVWSKPDSNLSLRADIHTTDSFSTLTVENCNRNDAGKYTLTVENNSGSKSIT

Score	Expect	Method	Identities	Positives	Gaps
42.0 bits(97)	1e-11	Compositional matrix adjust.	21/77(27%)	36/77(46%)	1/77(1%)

```
Query  1   PRLQLPRHLRQTIQKKVGEPVNLLIPFQGKPRPQVTWTKEGQPLAGEEVSIRNSPTDTIL  60
           P L +   + +QT    + G   + L I +QG+P P    W+K     L+      I   + + + L
Sbjct  1   PELDIDANFKQTHVVRAGASIRLFIAYQGRPTPTAVWSKPDSNLS-LRADIHTTDSFSTL  59

Query  61  FIRAARRVHSGTYQVTV  77
            +     R   +G Y +TV
Sbjct  60  TVENCNRNDAGKYTLTV  76
```

8, Ig, 6

PRLQLPRHLRQTIQKKVGEPVNLLIPFQGKPRPQVTWTKEGQPLAGEEVSIRNSPTDTILFIRAARRVHSGTYQVTVRIENMEDKATLVLQVVDK

11, 153, Ig, 137

PDLELADDLKKTVTIRAGASLRLMVSVSGRPPPVITWSKQGIDLASRAIIDTTESYSLLIVDKVNRYDAGKYTIEAENQSGKKSATVLVK

Score	Expect	Method	Identities	Positives	Gaps
50.8 bits(120)	6e-15	Compositional matrix adjust.	23/91(25%)	50/91(54%)	1/91(1%)

```
Query  1   PRLQLPRHLRQTIQKKVGEPVNLLIPFQGKPRPQVTWTKEGQPLAGEEVSIRNSPTDTIL  60
           P L+L    L++T+  + G   + L++    G+P P +TW+K+G  LA    + I   + + ++L
Sbjct  1   PDLELADDLKKTVTIRAGASLRLMVSVSGRPPPVITWSKQGIDLASRAI-IDTTESYSLL  59

Query  61  FIRAARRVHSGTYQVTVRIENMEDKATLVLQ  91
            +     R   +G Y +       ++ +  AT++++
Sbjct  60  IVDKVNRYDAGKYTIEAENQSGKKSATVLVK  90
```

MyBP-C cardiac (human) and titin (human)

9, Fn, 3

PPQDLRVTDAWGLNVALEWKPPQDVGNTELWGYTVQKADKKTMEWFTVLEHYRRTHCVVPELIIGNGYYFRVFSQNMVGFSDRAATTKEPVFIPRP

2, 63, Fn, 56

PPEKPEVSNVTKNTATVSWKRPVDDGGSEITGYHVERREKKSLRWVRAIKTPVSDLRCKVTGLQEGSTYEFRVSAENRAGIGPPSEASDSVLMKDA

Score	Expect	Method	Identities	Positives	Gaps
50.1 bits(118)	1e-14	Compositional matrix adjust.	25/81(31%)	41/81(50%)	1/81(1%)

```
Query  1    PPQDLRVTDAWGLNVALEWKPPQDVGNTELWGYTVQKADKKTMEWFTVLEH-YRRTHCVV  59
            PP+   V++       + WK P D G +E+ GY V++ +KK++ W   ++       C V
Sbjct  1    PPEKPEVSNVTKNTATVSWKRPVDDGGSEITGYHVERREKKSLRWVRAIKTPVSDLRCKV  60

Query  60   PELIIGNGYYFRVFSQNMVGF  80
              L  G+ Y FRV ++N  G
Sbjct  61   TGLQEGSTYEFRVSAENRAGI  81
```

9, Fn, 3

PPQDLRVTDAWGLNVALEWKPPQDVGNTELWGYTVQKADKKTMEWFTVLEHYRRTHCVVPELIIGNGYYFRVFSQNMVGFSDRAATTKEPVFIPRP

2, 64, Fn, 57

PPSNPHVTDTTKKSASLAWGKPHYDGGLEITGYVVEHQKVGDEAWIKDTTGTALRITQFVVPDLQTKEKYNFRISAINDAGVGEPAVIPDVEIVER

Score	Expect	Method	Identities	Positives	Gaps
42.0 bits(97)	2e-11	Compositional matrix adjust.	30/96(31%)	38/96(39%)	9/96(9%)

```
Query  1    PPQDLRVTDAWGLNVALEWKPPQDVGNTELWGYTVQKADKKTMEWF--TVLEHYRRTHCV  58
            PP +  VTD    + +L W  P   G  E+ GY V+        W   T     R T  V
Sbjct  1    PPSNPHVTDTTKKSASLAWGKPHYDGGLEITGYVVEHQKVGDEAWIKDTTGTALRITQFV  60

Query  59   VPELIIGNGYYFRVFSQNMVGFSDRAATTKEPVFIP  94
            VP+L     Y FR+ + N  G         EP  IP
Sbjct  61   VPDLQTKEKYNFRISAINDAGVG-------EPAVIP  89
```

MyBP-C cardiac (human) and titin (human)

9, Fn, 3

PPQDLRVTDAWGLNVALEWKPPQDVGNTELWGYTVQKADKKTMEWFTVLEHYRRTHCVVPELIIGNGYYFRVFSQNMVGFSDRAATTKEPVFIPRP

3, 75, Fn, 65

PPGNPRVLDTSRSSISIAWNKPIYDGGSEITGYMVEIALPEEDEWQIVTPPAGLKATSYTITGLTENQEYKIRIYAMNSEGLGEPALVPGTPKAEDR

Score	Expect	Method	Identities	Positives	Gaps
37.0 bits(84)	2e-09	Compositional matrix adjust.	25/96(26%)	40/96(41%)	9/96(9%)

```
Query  1    PPQDLRVTDAWGLNVALEWKPPQDVGNTELWGYTVQKADKKTMEWFTVL--EHYRRTHCV  58
            PP + RV D    ++++ W  P   G +E+ GY V+ A  +  EW  V      + T
Sbjct  1    PPGNPRVLDTSRSSISIAWNKPIYDGGSEITGYMVEIALPEEDEWQIVTPPAGLKATSYT  60

Query  59   VPELIIGNGYYFRVFSQNMVGFSDRAATTKEPVFIP  94
            +  L     Y  R+++ N  G         EP  +P
Sbjct  61   ITGLTENQEYKIRIYAMNSEGLG-------EPALVP  89
```

MyBP-C cardiac (human) and titin (human)

9, Fn, 3

PPQDLRVTDAWGLNVALEWKPPQDVGNTELWGYTVQKADKKTMEWFTVLEHYRRTHCVVPELIIGNGYYFRVFSQNMVGFSDRAATTKEPVFIPRP

4, 85, Fn, 72

APKAPEVTTVTKDSMIVVWERPASDGGSEILGYVLEKRDKEGIRWTRCHKRLIGELRLRVTGLIENHDYEFRVSAENAAGLSEPSPPSAYQKACDP

Score	Expect	Method	Identities	Positives	Gaps
40.0 bits(92)	1e-10	Compositional matrix adjust.	25/84(30%)	40/84(47%)	5/84(5%)

```
Query  2    PQDLRVTDAWGLNVALEWKPPQDVGNTELWGYTVQKADKKTMEWFTVLEHYR---RTHCV  58
            P+   VT      ++ + W+ P   G +E+ GY ++K DK+ + W     H R
Sbjct  2    PKAPEVTTVTKDSMIVVWERPASDGGSEILGYVLEKRDKEGIRWTRC--HKRLIGELRLR  59

Query  59   VPELIIGNGYYFRVFSQNMVGFSD  82
            V  LI  + Y FRV ++N  G S+
Sbjct  60   VTGLIENHDYEFRVSAENAAGLSE  83
```

9, Fn, 3

PPQDLRVTDAWGLNVALEWKPPQDVGNTELWGYTVQKADKKTMEWFTVLEHYRRTHCVVPELIIGNGYYFRVFSQNMVGFSDRAATTKEPVFIPRP

4, 86, Fn, 73

PPNNPKVIDITRSSVFLSWSKPIYDGGCEIQGYIVEKCDVSVGEWTMCTPPTGINKTNIEVEKLLEKHEYNFRICAINKAGVGEHADVPGPIIVEEK

Score	Expect	Method	Identities	Positives	Gaps
43.5 bits(101)	5e-12	Compositional matrix adjust.	27/86(31%)	40/86(46%)	2/86(2%)

```
Query  1    PPQDLRVTDAWGLNVALEWKPPQDVGNTELWGYTVQKADKKTMEW--FTVLEHYRRTHCV  58
            PP + +V D     +V L W  P   G  E+ GY V+K D    EW  T      +T+
Sbjct  1    PPNNPKVIDITRSSVFLSWSKPIYDGGCEIQGYIVEKCDVSVGEWTMCTPPTGINKTNIE  60

Query  59   VPELIIGNGYYFRVFSQNMVGFSDRA  84
            V +L+  + Y FR+ + N  G   + A
Sbjct  61   VEKLLEKHEYNFRICAINKAGVGEHA  86
```

MyBP-C cardiac (human) and titin (human)

9, Fn, 3

PPQDLRVTDAWGLNVALEWKPPQDVGNTELWGYTVQKADKKTMEWFTVLEHYRRTHCVVPELIIGNGYYFRVFSQNMVGFSDRAATTKEPVFIPRP

5, 96, Fn, 80

PPKSLEVTNIAKDSMTVCWNRPDSDGGSEIIGYIVEKRDRSGIRWIKCNKRRITDLRLRVTGLTEDHEYEFRVSAENAAGVGEPSPATVYYKACDP

Score	Expect	Method	Identities	Positives	Gaps
42.0 bits(97)	2e-11	Compositional matrix adjust.	26/90(29%)	42/90(46%)	5/90(5%)

```
Query  1    PPQDLRVTDAWGLNVALEWKPPQDVGNTELWGYTVQKADKKTMEWFTVLEHYRRT---HC  57
            PP+ L VT+    ++ + W  P   G +E+ GY V+K D+  + W     + RR
Sbjct  1    PPKSLEVTNIAKDSMTVCWNRPDSDGGSEIIGYIVEKRDRSGIRWIKC--NKRRITDLRL  58

Query  58   VVPELIIGNGYYFRVFSQNMVGFSDRAATT  87
             V  L   + Y FRV ++N  G  + +  T
Sbjct  59   RVTGLTEDHEYEFRVSAENAAGVGEPSPAT  88
```

9, Fn, 3

PPQDLRVTDAWGLNVALEWKPPQDVGNTELWGYTVQKADKKTMEWFTVLEHYRRTHCVVPELIIGNGYYFRVFSQNMVGFSDRAATTKEPVFIPRP

5, 97, Fn, 81

PPTNAHIVDTTKNSITLAWGKPIYDGGSEILGYVVEICKADEEEWQIVTPQTGLRVTRFEISKLTEHQEYKIRVCALNKVGLGEATSVPGTVKPEDK

Score	Expect	Method	Identities	Positives	Gaps
37.7 bits(86)	8e-10	Compositional matrix adjust.	24/87(28%)	39/87(44%)	2/87(2%)

```
Query  1    PPQDLRVTDAWGLNVALEWKPPQDVGNTELWGYTVQ--KADKKTMEWFTVLEHYRRTHCV  58
            PP +  + D    ++ L W  P   G +E+ GY V+  KAD++  +  T     R T
Sbjct  1    PPTNAHIVDTTKNSITLAWGKPIYDGGSEILGYVVEICKADEEEWQIVTPQTGLRVTRFE  60

Query  59   VPELIIGNGYYFRVFSQNMVGFSDRAA  85
            + +L     Y  RV + N VG  +  +
Sbjct  61   ISKLTEHQEYKIRVCALNKVGLGEATS  87
```

MyBP-C cardiac (human) and titin (human)

9, Fn, 3

PPQDLRVTDAWGLNVALEWKPPQDVGNTELWGYTVQKADKKTMEWFTVLEHYRRTHCVVPELIIGNGYYFRVFSQNMVGFSDRAATTKEPVFIPRP

6, 107, Fn, 88

PPSTPEVSAITKDSMVVTWARPVDDGGTEIEGYILEKRDKEGVRWTKCNKKTLTDLRLRVTGLTEGHSYEFRVAAENAAGVGEPSEPSVFYRACDA

Score	Expect	Method	Identities	Positives	Gaps
40.0 bits(92)	9e-11	Compositional matrix adjust.	24/83(29%)	38/83(45%)	1/83(1%)

```
Query  1   PPQDLRVTDAWGLNVALEWKPPQDVGNTELWGYTVQKADKKTMEWFTVLEH-YRRTHCVV  59
           PP    V+     ++ + W  P D G TE+ GY ++K DK+ + W     +        V
Sbjct  1   PPSTPEVSAITKDSMVVTWARPVDDGGTEIEGYILEKRDKEGVRWTKCNKKTLTDLRLRV  60

Query  60  PELIIGNGYYFRVFSQNMVGFSD  82
             L  G+ Y FRV ++N  G  +
Sbjct  61  TGLTEGHSYEFRVAAENAAGVGE  83
```

9, Fn, 3

PPQDLRVTDAWGLNVALEWKPPQDVGNTELWGYTVQKADKKTMEWFTVLEHYRRTHCVVPELIIGNGYYFRVFSQNMVGFSDRAATTKEPVFIPRP

6, 108, Fn, 89

PPSNPKVTDTSRSSVSLAWSKPIYDGGAPVKGYVVEVKEAAADEWTTCTPPTGLQGKQFTVTKLKENTEYNFRICAINSEGVGEPATLPGSVVAQER

Score	Expect	Method	Identities	Positives	Gaps
35.0 bits(79)	9e-09	Compositional matrix adjust.	24/86(28%)	36/86(41%)	2/86(2%)

```
Query  1   PPQDLRVTDAWGLNVALEWKPPQDVGNTELWGYTVQKADKKTMEWFTVL--EHYRRTHCV  58
           PP + +VTD    +V+L W  P   G   + GY V+  +    EW T       +
Sbjct  1   PPSNPKVTDTSRSSVSLAWSKPIYDGGAPVKGYVVEVKEAAADEWTTCTPPTGLQGKQFT  60

Query  59  VPELIIGNGYYFRVFSQNMVGFSDRA  84
           V +L     Y FR+ + N  G  + A
Sbjct  61  VTKLKENTEYNFRICAINSEGVGEPA  86
```

MyBP-C cardiac (human) and titin (human)

9, Fn, 3

PPQDLRVTDAWGLNVALEWKPPQDVGNTELWGYTVQKADKKTMEWFTVLEHYRRTHCVVPELIIGNGYYFRVFSQNMVGFSDRAATTKEPVFIPRP

7, 118, Fn, 96

PPTSLEITSVTKESMTLCWSRPESDGGSEISGYIIERREKNSLRWVRVNKKPVYDLRVKSTGLREGCEYEYRVYAENAAGLSLPSETSPLIRAEDP

Score	Expect	Method	Identities	Positives	Gaps
43.1 bits(100)	8e-12	Compositional matrix adjust.	22/82(27%)	39/82(47%)	1/82(1%)

```
Query  1   PPQDLRVTDAWGLNVALEWKPPQDVGNTELWGYTVQKADKKTMEWFTVLEH-YRRTHCVV  59
           PP  L +T     ++ L W  P+  G +E+ GY +++ +K ++ W  V +
Sbjct  1   PPTSLEITSVTKESMTLCWSRPESDGGSEISGYIIERREKNSLRWVRVNKKPVYDLRVKS  60

Query  60  PELIIGNGYYFRVFSQNMVGFS  81
             L  G  Y +RV+++N  G S
Sbjct  61  TGLREGCEYEYRVYAENAAGLS  82
```

9, Fn, 3

PPQDLRVTDAWGLNVALEWKPPQDVGNTELWGYTVQKADKKTMEWFTVLEHYRRTHCVVPELIIGNGYYFRVFSQNMVGFSDRAATTKEPVFIPRP

7, 119, Fn, 97

PPSKPKIVDSGKTTITIAWVKPLFDGGAPITGYTVEYKKSDDTDWKTSIQSLRGTEYTISGLTTGAEYVFRVKSVNKVGASDPSDSSDPQIAKER

Score	Expect	Method	Identities	Positives	Gaps
50.1 bits(118)	1e-14	Compositional matrix adjust.	26/82(32%)	37/82(45%)	0/82(0%)

```
Query  1   PPQDLRVTDAWGLNVALEWKPPQDVGNTELWGYTVQKADKKTMEWFTVLEHYRRTHCVVP  60
           PP   ++ D+    + + W  P   G   + GYTV+       +W T ++  R T   +
Sbjct  1   PPSKPKIVDSGKTTITIAWVKPLFDGGAPITGYTVEYKKSDDTDWKTSIQSLRGTEYTIS  60

Query  61  ELIIGNGYYFRVFSQNMVGFSD  82
            L  G  Y FRV S N VG SD
Sbjct  61  GLTTGAEYVFRVKSVNKVGASD  82
```

MyBP-C cardiac (human) and titin (human)

9, Fn, 3

PPQDLRVTDAWGLNVALEWKPPQDVGNTELWGYTVQKADKKTMEWFTVLEHYRRTHCVVPELIIGNGYYFRVFSQNMVGFSDRAATTKEPVFIPRP

8, 129, Fn, 104

PPGIPEVTKITKNSMTVVWSRPIADGGSDISGYFLEKRDKKSLGWFKVLKETIRDTRQKVTGLTENSDYQYRVCAVNAAGQGPFSEPSEFYKAADP

Score	Expect	Method	Identities	Positives	Gaps
43.5 bits(101)	5e-12	Compositional matrix adjust.	27/80(34%)	40/80(50%)	1/80(1%)

```
Query  1   PPQDLRVTDAWGLNVALEWKPPQDVGNTELWGYTVQKADKKTMEWFTVL-EHYRRTHCVV  59
           PP    VT     ++ + W  P   G +++ GY ++K DKK++ WF VL E  R T   V
Sbjct  1   PPGIPEVTKITKNSMTVVWSRPIADGGSDISGYFLEKRDKKSLGWFKVLKETIRDTRQKV  60

Query  60  PELIIGNGYYFRVFSQNMVG  79
             L   + Y +RV + N  G
Sbjct  61  TGLTENSDYQYRVCAVNAAG  80
```

9, Fn, 3

PPQDLRVTDAWGLNVALEWKPPQDVGNTELWGYTVQKADKKTMEWFTVLEHYRRTHCVVPELIIGNGYYFRVFSQNMVGFSDRAATTKEPVFIPRP

8, 130, Fn, 105

PPAKIRIADSTKSSITLGWSKPVYDGGSAVTGYVVEIRQGEEEEWTTVSTKGEVRTTEYVVSNLKPGVNYYFRVSAVNCAGQGEPIEMNEPVQAKDI

Score	Expect	Method	Identities	Positives	Gaps
47.0 bits(110)	2e-13	Compositional matrix adjust.	28/81(35%)	38/81(46%)	2/81(2%)

```
Query  1   PPQDLRVTDAWGLNVALEWKPPQDVGNTELWGYTVQKADKKTMEWFTVLE--HYRRTHCV  58
           PP  +R+ D+   ++ L W  P   G + + GY V+    +  EW TV      R T  V
Sbjct  1   PPAKIRIADSTKSSITLGWSKPVYDGGSAVTGYVVEIRQGEEEEWTTVSTKGEVRTTEYV  60

Query  59  VPELIIGNGYYFRVFSQNMVG  79
           V  L  G  YYFRV + N  G
Sbjct  61  VSNLKPGVNYYFRVSAVNCAG  81
```

MyBP-C cardiac (human) and titin (human)

9, Fn, 3

PPQDLRVTDAWGLNVALEWKPPQDVGNTELWGYTVQKADKKTMEWFTVLEHYRRTHCVVPELIIGNGYYFRVFSQNMVGFSDRAATTKEPVFIPRP

9, 140, Fn, 112

APGIPEPSNITGNSITLTWARPESDGGSEIQQYILERREKKSTRWVKVISKRPISETRFKVTGLTEGNEYEFHVMAENAAGVGPASGISRLIKCREP

Score	Expect	Method	Identities	Positives	Gaps
42.0 bits(97)	2e-11	Compositional matrix adjust.	21/83(25%)	39/83(46%)	2/83(2%)

```
Query  8   TDAWGLNVALEWKPPQDVGNTELWGYTVQKADKKTMEWFTVLEH--YRRTHCVVPELIIG  65
           ++  G ++ L W  P+  G +E+  Y +++ +KK+  W  V+       T   V  L  G
Sbjct  8   SNITGNSITLTWARPESDGGSEIQQYILERREKKSTRWVKVISKRPISETRFKVTGLTEG  67

Query  66  NGYYFRVFSQNMVGFSDRAATTK  88
           N Y F V ++N  G    +  ++
Sbjct  68  NEYEFHVMAENAAGVGPASGISR  90
```

9, Fn, 3

PPQDLRVTDAWGLNVALEWKPPQDVGNTELWGYTVQKADKKTMEWFTVLEHYRRTHCVVPELIIGNGYYFRVFSQNMVGFSDRAATTKEPVFIPRP

9, 141, Fn, 113

PPGPPTVVKVTDTSKTTVSLEWSKPVFDGGMEIIGYIIEMCKADLGDWHKVNAEACVKTRYTVTDLQAGEEYKFRVSAINGAGKGDSCEVTGTIKAVDR

Score	Expect	Method	Identities	Positives	Gaps
47.4 bits(111)	2e-13	Compositional matrix adjust.	29/88(33%)	39/88(44%)	1/88(1%)

```
Query  1   PPQDLRVTDAWGLNVALEWKPPQDVGNTELWGYTVQKADKKTMEWFTV-LEHYRRTHCVV  59
           PP  ++VTD     V+LEW  P   G  E+ GY ++       +W  V  E   +T   V
Sbjct  4   PPTVVKVTDTSKTTVSLEWSKPVFDGGMEIIGYIIEMCKADLGDWHKVNAEACVKTRYTV  63

Query  60  PELIIGNGYYFRVFSQNMVGFSDRAATT  87
            +L  G  Y FRV + N  G  D    T
Sbjct  64  TDLQAGEEYKFRVSAINGAGKGDSCEVT  91
```

MyBP-C cardiac (human) and titin (human)

9, Fn, 3

PPQDLRVTDAWGLNVALEWKPPQDVGNTELWGYTVQKADKKTMEWFTVLEHYRRTHCVVPELIIGNGYYFRVFSQNMVGFSDRAATTKEPVFIPRP

10, 151, Fn, 120

PPGIPEEVGTGKEHIIIQWTKPESDGGNEISNYLVDKREKKSLRWTRVNKDYVVYDTRLKVTSLMEGCDYQFRVTAVNAAGNSEPSEASNFISCREP

Score	Expect	Method	Identities	Positives	Gaps
42.0 bits(97)	2e-11	Compositional matrix adjust.	23/71(32%)	36/71(50%)	2/71(2%)

```
Query  14   NVALEWKPPQDVGNTELWGYTVQKADKKTMEWFTVLEHY--RRTHCVVPELIIGNGYYFR  71
            ++ ++W  P+  G  E+  Y V K +KK++ W  V + Y    T   V  L+ G  Y FR
Sbjct  14   HIIIQWTKPESDGGNEISNYLVDKREKKSLRWTRVNKDYVVYDTRLKVTSLMEGCDYQFR  73

Query  72   VFSQNMVGFSD  82
            V + N  G S+
Sbjct  74   VTAVNAAGNSE  84
```

9, Fn, 3

PPQDLRVTDAWGLNVALEWKPPQDVGNTELWGYTVQKADKKTMEWFTVLEHYRRTHCVVPELIIGNGYYFRVFSQNMVGFSDRAATTKEPVFIPRP

10, 152, Fn, 121

PPSAPRVVDTTKHSISLAWTKPMYDGGTDIVGYVLEMQEKDTDQWYRVHTNATIRNTEFTVPDLKMGQKYSFRVAAVNVKGMSEYSESIAEIEPVER

Score	Expect	Method	Identities	Positives	Gaps
55.5 bits(132)	1e-16	Compositional matrix adjust.	30/91(33%)	48/91(52%)	2/91(2%)

```
Query  1    PPQDLRVTDAWGLNVALEWKPPQDVGNTELWGYTVQKADKKTMEWFTVLEH--YRRTHCV  58
            PP   RV D    +++L W   P   G T++ GY ++  +K T +W+ V  +   R T
Sbjct  1    PPSAPRVVDTTKHSISLAWTKPMYDGGTDIVGYVLEMQEKDTDQWYRVHTNATIRNTEFT  60

Query  59   VPELIIGNGYYFRVFSQNMVGFSDRAATTKE  89
            VP+L +G  Y FRV + N+ G S+ + +  E
Sbjct  61   VPDLKMGQKYSFRVAAVNVKGMSEYSESIAE  91
```

MyBP-C cardiac (human) and titin (human)

10, Ig, 7

PSFTQPLVNRSVIAGYTAMLCCAVRGSPKPKISWFKNGLDLGEDARFRMFSKQGVLTLEIRKPCPFDGGIYVCRATNLQGEARCECRLEVRVPQ

3, 65, Ig, 114

PDFELDAELRRTLVVRAGLSIRIFVPIKGRPAPEVTWTKDNINLKNRANIENTESFTLLIIPECNRYDTGKFVMTIENPAGKKSGFVNVRVLD

Score	Expect	Method	Identities	Positives	Gaps
30.0 bits(66)	7e-07	Compositional matrix adjust.	19/71(27%)	32/71(45%)	4/71(5%)

```
Query  12  VIAGYTAMLCCAVRGSPKPKISWFKNGLDLGEDARFRMFSKQGVLTLEIRKPCP-FDGGI  70
            V AG +  +   ++G P P+++W K+ ++L   A           TL I   C  +D G
Sbjct  15  VRAGLSIRIFVPIKGRPAPEVTWTKDNINLKNRAN---IENTESFTLLIIPECNRYDTGK  71

Query  71  YVCRATNLQGE  81
            +V    N  G+
Sbjct  72  FVMTIENPAGK  82
```

10, Ig, 7

PSFTQPLVNRSVIAGYTAMLCCAVRGSPKPKISWFKNGLDLGEDARFRMFSKQGVLTLEIRKPCPFDGGIYVCRATNLQGEARCECRLEVRVPQ

4, 76, Ig, 117

PEIELDADLRKVVTIRACCTLRLFVPIKGRPAPEVKWARDHGESLDKASIESTSSYTLLIVGNVNRFDSGKYILTVENSSGSKSAFVNVR

Score	Expect	Method	Identities	Positives	Gaps
25.4 bits(54)	3e-05	Compositional matrix adjust.	15/64(23%)	26/64(40%)	2/64(3%)

```
Query  17  TAMLCCAVRGSPKPKISWFKNGLDLGEDARFRMFSKQGVLTLEIRKPCPFDGGIYVCRAT  76
            T  L   ++G P P++ W ++  +  + A     S   +L   +     FD G Y+
Sbjct  20  TLRLFVPIKGRPAPEVKWARDHGESLDKASIESTSSYTLLI--VGNVNRFDSGKYILTVE  77

Query  77  NLQG  80
            N  G
Sbjct  78  NSSG  81
```

MyBP-C cardiac (human) and titin (human)

10, Ig, 7

PSFTQPLVNRSVIAGYTAMLCCAVRGSPKPKISWFKNGLDLGEDARFRMFSKQGVLTLEIRKPCPFDGGIYVCRATNLQGEARCECRLEVRVPQ

5, 87, Ig, 120

PDIDLDLELRKIINIRAGGSLRLFVPIKGRPTPEVKWGKVDGEIRDAAIIDVTSSFTSLVLDNVNRYDSGKYTLTLENSSGTKSAFVT

Score	Expect	Method	Identities	Positives	Gaps
24.6 bits(52)	5e-05	Compositional matrix adjust.	17/67(25%)	28/67(41%)	2/67(2%)

```
Query  14  AGYTAMLCCAVRGSPKPKISWFKNGLDLGEDARFRMFSKQGVLTLEIRKPCPFDGGIYVC  73
            AG +  L   ++G P P++ W K   ++ + A   + S    L L+      +D G Y
Sbjct  17  AGGSLRLFVPIKGRPTPEVKWGKVDGEIRDAAIIDVTSSFTSLVLD--NVNRYDSGKYTL  74

Query  74  RATNLQG  80
               N  G
Sbjct  75  TLENSSG  81
```

10, Ig, 7

PSFTQPLVNRSVIAGYTAMLCCAVRGSPKPKISWFKNGLDLGEDARFRMFSKQGVLTLEIRKPCPFDGGIYVCRATNLQGEARCECRLEVRVPQ

6, 98, Ig, 123

PELDLDSELRKGIVVRAGGSARIHIPFKGRPTPEITWSREEGEFTDKVQIEKGVNYTQLSIDNCDRNDAGKYILKLENSSGSKSAFVTVK

Score	Expect	Method	Identities	Positives	Gaps
24.6 bits(52)	6e-05	Compositional matrix adjust.	20/73(27%)	33/73(45%)	10/73(13%)

```
Query  12  VIAGYTAMLCCAVRGSPKPKISWFKNGLDLGEDARF--RMFSKQGV--LTLEIRKPCPFD  67
            V AG +A +    +G P P+I+W +      E+  F  ++  ++GV    L I      D
Sbjct  15  VRAGGSARIHIPFKGRPTPEITWSR------EEGEFTDKVQIEKGVNYTQLSIDNCDRND  68

Query  68  GGIYVCRATNLQG  80
             G Y+ +  N  G
Sbjct  69  AGKYILKLENSSG  81
```

MyBP-C cardiac (human) and titin (human)

10, Ig, 7

PSFTQPLVNRSVIAGYTAMLCCAVRGSPKPKISWFKNGLDLGEDARFRMFSKQGVLTLEIRKPCPFDGGIYVCRATNLQGEARCECRLEVRVPQ

7, 109, Ig, 126

PEIELDADLRKVVVLRASATLRLFVTIKGRPEPEVKWEKAEGILTDRAQIEVTSSFTMLVIDNVTRFDSGRYNLTLENNSGSKTAFVNVR

Score	Expect	Method	Identities	Positives	Gaps
28.9 bits(63)	2e-06	Compositional matrix adjust.	18/67(27%)	29/67(43%)	2/67(2%)

```
Query  14   AGYTAMLCCAVRGSPKPKISWFKNGLDLGEDARFRMFSKQGVLTLEIRKPCPFDGGIYVC  73
            A  T  L    ++G P+P++ W K     L + A+   + S   +L ++        FD G Y
Sbjct  17   ASATLRLFVTIKGRPEPEVKWEKAEGILTDRAQIEVTSSFTMLVID--NVTRFDSGRYNL  74

Query  74   RATNLQG  80
              N  G
Sbjct  75   TLENNSG  81
```

10, Ig, 7

PSFTQPLVNRSVIAGYTAMLCCAVRGSPKPKISWFKNGLDLGEDARFRMFSKQGVLTLEIRKPCPFDGGIYVCRATNLQGEARCECRLEVRVPQ

8, 120, Ig, 129

PQIAKEREEEPLFDIDSEMRKTLIVKAGASFTMTVPFRGRPVPNVLWSKPDTDLRTRAYVDTTDSRTSLTIENANRNDSGKYTLTIQNVLSAASLT

Score	Expect	Method	Identities	Positives	Gaps
27.7 bits(60)	4e-06	Compositional matrix adjust.	20/71(28%)	26/71(36%)	2/71(2%)

```
Query  12   VIAGYTAMLCCAVRGSPKPKISWFKNGLDLGEDARFRMFSKQGVLTLEIRKPCPFDGGIY  71
            V AG +   +     RG P P + W K    DL    A          +  LT+E         D G Y
Sbjct  25   VKAGASFTMTVPFRGRPVPNVLWSKPDTDLRTRAYVDTTDSRTSLTIE--NANRNDSGKY  82

Query  72   VCRATNLQGEA  82
                 N+   A
Sbjct  83   TLTIQNVLSAA  93
```

MyBP-C cardiac (human) and titin (human)

10, Ig, 7

PSFTQPLVNRSVIAGYTAMLCCAVRGSPKPKISWFKNGLDLGEDARFRMFSKQGVLTLEIRKPCPFDGGIYVCRATNLQGEARCECRLEVRVPQ

9, 131, Ig, 131

PEIDLDVALRTSVIAKAGEDVQVLIPFKGRPPPTVTWRKDEKNLGSDARYSIENTDSSSLLTIPQVTRNDTGKYILTIENGVGEPKSSTVS

Score	Expect	Method	Identities	Positives	Gaps
39.7 bits(91)	1e-10	Compositional matrix adjust.	20/73(27%)	32/73(43%)	0/73(0%)

```
Query  14  AGYTAMLCCAVRGSPKPKISWFKNGLDLGEDARFRMFSKQGVLTLEIRKPCPFDGGIYVC  73
            AG    +    +G P P ++W K+  +LG DAR+ + +      L I +    D G Y+
Sbjct  17  AGEDVQVLIPFKGRPPPTVTWRKDEKNLGSDARYSIENTDSSSLLTIPQVTRNDTGKYIL  76

Query  74  RATNLQGEARCEC  86
               N  GE +
Sbjct  77  TIENGVGEPKSST  89
```

MyBP-C cardiac (human) and titin (human)

10, Ig, 7

PSFTQPLVNRSVIAGYTAMLCCAVRGSPKPKISWFKNGLDLGEDARFRMFSKQGVLTLEIRKPCPFDGGIYVCRATNLQGEARCECRLEVRVPQ

9, 141, Fn, 113

PPGPPTVVKVTDTSKTTVSLEWSKPVFDGGMEIIGYIIEMCKADLGDWHKVNAEACVKTRYTVTDLQAGEEYKFRVSAINGAGKGDSCEVTGTIKAVDR

Score	Expect	Method	Identities	Positives	Gaps
17.7 bits(34)	0.019	Compositional matrix adjust.	14/60(23%)	26/60(43%)	8/60(13%)

```
Query  3    FTQPLVNRSV-IAGYTAMLCCAVRGSPKP-------KISWFKNGLDLGEDARFRMFSKQG  54
            +++P+ +  +  I GY   +C A  G           K  +    L  GE+ +FR+ +  G
Sbjct  22   WSKPVFDGGMEIIGYIIEMCKADLGDWHKVNAEACVKTRYTVTDLQAGEEYKFRVSAING  81
```

10, Ig, 7

PSFTQPLVNRSVIAGYTAMLCCAVRGSPKPKISWFKNGLDLGEDARFRMFSKQGVLTLEIRKPCPFDGGIYVCRATNLQGEARCECRLEVRVPQ

10, 142, Ig, 134

PELDIDANFKQTHVVRAGASIRLFIAYQGRPTPTAVWSKPDSNLSLRADIHTTDSFSTLTVENCNRNDAGKYTLTVENNSGSKSIT

Score	Expect	Method	Identities	Positives	Gaps
25.0 bits(53)	4e-05	Compositional matrix adjust.	19/69(28%)	25/69(36%)	2/69(2%)

```
Query  12   VIAGYTAMLCCAVRGSPKPKISWFKNGLDLGEDARFRMFSKQGVLTLEIRKPCPFDGGIY  71
            V AG +  L  A +G P P   W K   +L    R  + +     TL +      D G Y
Sbjct  15   VRAGASIRLFIAYQGRPTPTAVWSKPDSNLS--LRADIHTTDSFSTLTVENCNRNDAGKY  72

Query  72   VCRATNLQG  80
                 N   G
Sbjct  73   TLTVENNSG  81
```

MyBP-C cardiac (human) and titin (human)

10, Ig, 7

PSFTQPLVNRSVIAGYTAMLCCAVRGSPKPKISWFKNGLDLGEDARFRMFSKQGVLTLEIRKPCPFDGGIYVCRATNLQGEARCECRLEVRVPQ

11, 153, Ig, 137

PDLELADDLKKTVTIRAGASLRLMVSVSGRPPPVITWSKQGIDLASRAIIDTTESYSLLIVDKVNRYDAGKYTIEAENQSGKKSATVLVK

Score	Expect	Method	Identities	Positives	Gaps
39.7 bits(91)	1e-10	Compositional matrix adjust.	22/68(32%)	31/68(45%)	2/68(2%)

```
Query  14  AGYTAMLCCAVRGSPKPKISWFKNGLDLGEDARFRMFSKQGVLTLEIRKPCPFDGGIYVC  73
            AG +  L  +V G P P I+W K G+DL   A         +L ++  K   +D G Y
Sbjct  17  AGASLRLMVSVSGRPPPVITWSKQGIDLASRAIIDTTESYSLLIVD--KVNRYDAGKYTI  74

Query  74  RATNLQGE  81
             A N  G+
Sbjct  75  EAENQSGK  82
```

MyBP-C fast skeletal (human) and titin (human)

8, Ig, 6

PKIRLPRHLRQTYIRKVGEQLNLVVPFQGKPRPQVVWTKGGAPLDTSRVHVRTSDFDTVFFVRQAARSDSGEYELSVQIENMKDTATIRIRVVEK

3, 65, Ig, 114

PDFELDAELRRTLVVRAGLSIRIFVPIKGRPAPEVTWTKDNINLKNRANIENTESFTLLIIPECNRYDTGKFVMTIENPAGKKSGFVNVRVLD

Score	Expect	Method	Identities	Positives	Gaps
53.9 bits(128)	4e-16	Compositional matrix adjust.	24/94(26%)	52/94(55%)	1/94(1%)

```
Query  1    PKIRLPRHLRQTYIRKVGEQLNLVVPFQGKPRPQVVWTKGGAPLDTSRVHVRTSDFDTVF  60
            P   L   LR+T + + G  + + VP +G+P P+V WTK    L  +R ++  ++  T+
Sbjct  1    PDFELDAELRRTLVVRAGLSIRIFVPIKGRPAPEVTWTKDNINLK-NRANIENTESFTLL  59

Query  61   FVRQAARSDSGEYELSVQIENMKDTATIRIRVVE  94
             + +  R D+G++ ++++    K +  + +RV++
Sbjct  60   IIPECNRYDTGKFVMTIENPAGKKSGFVNVRVLD  93
```

8, Ig, 6

PKIRLPRHLRQTYIRKVGEQLNLVVPFQGKPRPQVVWTKGGAPLDTSRVHVRTSDFDTVFFVRQAARSDSGEYELSVQIENMKDTATIRIRVVEK

4, 76, Ig, 117

PEIELDADLRKVVTIRACCTLRLFVPIKGRPAPEVKWARDHGESLDKASIESTSSYTLLIVGNVNRFDSGKYILTVENSSGSKSAFVNVR

Score	Expect	Method	Identities	Positives	Gaps
48.9 bits(115)	3e-14	Compositional matrix adjust.	30/92(33%)	48/92(52%)	3/92(3%)

```
Query  1    PKIRLPRHLRQTYIRKVGEQLNLVVPFQGKPRPQVVWTKG-GAPLDTSRVHVRTSDFDTV  59
            P+I L   LR+    +    L L VP +G+P P+V W +  G  LD + +   TS + T+
Sbjct  1    PEIELDADLRKVVTIRACCTLRLFVPIKGRPAPEVKWARDHGESLDKASIE-STSSY-TL  58

Query  60   FFVRQAARSDSGEYELSVQIENMKDTATIRIR  91
              V    R DSG+Y L+V+    + +A + +R
Sbjct  59   LIVGNVNRFDSGKYILTVENSSGSKSAFVNVR  90
```

MyBP-C fast skeletal (human) and titin (human)

8, Ig, 6

PKIRLPRHLRQTYIRKVGEQLNLVVPFQGKPRPQVVWTKGGAPLDTSRVHVRTSDFDTVFFVRQAARSDSGEYELSVQIENMKDTATIRIRVVEK

5, 87, Ig, 120

PDIDLDLELRKIINIRAGGSLRLFVPIKGRPTPEVKWGKVDGEIRDAAIIDVTSSFTSLVLDNVNRYDSGKYTLTLENSSGTKSAFVT

Score	Expect	Method	Identities	Positives	Gaps
45.1 bits(105)	8e-13	Compositional matrix adjust.	26/78(33%)	39/78(50%)	1/78(1%)

```
Query  1    PKIRLPRHLRQTYIRKVGEQLNLVVPFQGKPRPQVVWTKGGAPLDTSRVHVRTSDFDTVF  60
            P I L   LR+    + G  L L VP +G+P P+V W K     +  + +    TS F T
Sbjct  1    PDIDLDLELRKIINIRAGGSLRLFVPIKGRPTPEVKWGKVDGEIRDAAIIDVTSSF-TSL  59

Query  61   FVRQAARSDSGEYELSVQ  78
             +     R DSG+Y L+++
Sbjct  60   VLDNVNRYDSGKYTLTLE  77
```

8, Ig, 6

PKIRLPRHLRQTYIRKVGEQLNLVVPFQGKPRPQVVWTKGGAPLDTSRVHVRTSDFDTVFFVRQAARSDSGEYELSVQIENMKDTATIRIRVVEK

6, 98, Ig, 123

PELDLDSELRKGIVVRAGGSARIHIPFKGRPTPEITWSREEGEFTDKVQIEKGVNYTQLSIDNCDRNDAGKYILKLENSSGSKSAFVTVK

Score	Expect	Method	Identities	Positives	Gaps
43.5 bits(101)	3e-12	Compositional matrix adjust.	20/91(22%)	46/91(50%)	1/91(1%)

```
Query  1    PKIRLPRHLRQTYIRKVGEQLNLVVPFQGKPRPQVVWTKGGAPLDTSRVHVRTSDFDTVF  60
            P++ L    LR+  + + G    + +PF+G+P P++ W++        T +V +       T
Sbjct  1    PELDLDSELRKGIVVRAGGSARIHIPFKGRPTPEITWSREEGEF-TDKVQIEKGVNYTQL  59

Query  61   FVRQAARSDSGEYELSVQIENMKDTATIRIR  91
             +     R+D+G+Y L ++  +    +A + ++
Sbjct  60   SIDNCDRNDAGKYILKLENSSGSKSAFVTVK  90
```

MyBP-C fast skeletal (human) and titin (human)

8, Ig, 6

PKIRLPRHLRQTYIRKVGEQLNLVVPFQGKPRPQVVWTKGGAPLDTSRVHVRTSDFDTVFFVRQAARSDSGEYELSVQIENMKDTATIRIRVVEK

6, 108, Fn, 89

PPSNPKVTDTSRSSVSLAWSKPIYDGGAPVKGYVVEVKEAAADEWTTCTPPTGLQGKQFTVTKLKENTEYNFRICAINSEGVGEPATLPGSVVAQER

Score	Expect	Method	Identities	Positives	Gaps
17.7 bits(34)	0.017	Compositional matrix adjust.	9/28(32%)	14/28(50%)	4/28(14%)

```
Query  34   QVVWTK----GGAPLDTSRVHVRTSDFD  57
             + W+K    GGAP+    V V+ +  D
Sbjct  16   SLAWSKPIYDGGAPVKGYVVEVKEAAAD  43
```

8, Ig, 6

PKIRLPRHLRQTYIRKVGEQLNLVVPFQGKPRPQVVWTKGGAPLDTSRVHVRTSDFDTVFFVRQAARSDSGEYELSVQIENMKDTATIRIRVVEK

7, 109, Ig, 126

PEIELDADLRKVVVLRASATLRLFVTIKGRPEPEVKWEKAEGILTDRAQIEVTSSFTMLVIDNVTRFDSGRYNLTLENNSGSKTAFVNVR

Score	Expect	Method	Identities	Positives	Gaps
51.2 bits(121)	3e-15	Compositional matrix adjust.	27/91(30%)	44/91(48%)	1/91(1%)

```
Query  1    PKIRLPRHLRQTYIRKVGEQLNLVVPFQGKPRPQVVWTKGGAPLDTSRVHVRTSDFDTVF  60
            P+I L   LR+  + +    L L V  +G+P P+V W K    L T R  +  +   T+
Sbjct  1    PEIELDADLRKVVVLRASATLRLFVTIKGRPEPEVKWEKAEGIL-TDRAQIEVTSSFTML  59

Query  61   FVRQAARSDSGEYELSVQIENMKDTATIRIR  91
            +     R DSG Y L+++  +   TA + +R
Sbjct  60   VIDNVTRFDSGRYNLTLENNSGSKTAFVNVR  90
```

MyBP-C fast skeletal (human) and titin (human)

8, Ig, 6

PKIRLPRHLRQTYIRKVGEQLNLVVPFQGKPRPQVVWTKGGAPLDTSRVHVRTSDFDTVFFVRQAARSDSGEYELSVQIENMKDTATIRIRVVEK

7, 119, Fn, 97

PPSKPKIVDSGKTTITIAWVKPLFDGGAPITGYTVEYKKSDDTDWKTSIOSLRGTEYTISGLTTGAEYVFRVKSVNKVGASDPSDSSDPQIAKER

Score	Expect	Method	Identities	Positives	Gaps
19.6 bits(39)	0.004	Compositional matrix adjust.	11/32(34%)	14/32(43%)	4/32(12%)

```
Query  28  QGKPRPQVVWTK----GGAPLDTSRVHVRTSD  55
             GK    + W K    GGAP+    V  + SD
Sbjct  10  SGKTTITIAWVKPLFDGGAPITGYTVEYKKSD  41
```

8, Ig, 6

PKIRLPRHLRQTYIRKVGEQLNLVVPFQGKPRPQVVWTKGGAPLDTSRVHVRTSDFDTVFFVRQAARSDSGEYELSVQIENMKDTATIRIRVVEK

8, 120, Ig, 129

PQIAKEREEEPLFDIDSEMRKTLIVKAGASFTMTVPFRGRPVPNVLWSKPDTDLRTRAYVDTTDSRTSLTIENANRNDSGKYTLTIQNVLSAASLT

Score	Expect	Method	Identities	Positives	Gaps
62.4 bits(150)	2e-19	Compositional matrix adjust.	30/78(38%)	45/78(57%)	1/78(1%)

```
Query  1    PKIRLPRHLRQTYIRKVGEQLNLVVPFQGKPRPQVVWTKGGAPLDTSRVHVRTSDFDTVF  60
            P   +   +R+T I K G    + VPF+G+P P V+W+K    L T R +V T+D  T
Sbjct  11   PLFDIDSEMRKTLIVKAGASFTMTVPFRGRPVPNVLWSKPDTDLRT-RAYVDTTDSRTSL  69

Query  61   FVRQAARSDSGEYELSVQ  78
             +  A R+DSG+Y L++Q
Sbjct  70   TIENANRNDSGKYTLTIQ  87
```

MyBP-C fast skeletal (human) and titin (human)

8, Ig, 6

PKIRLPRHLRQTYIRKVGEQLNLVVPFQGKPRPQVVWTKGGAPLDTSRVHVRTSDFDTVFFVRQAARSDSGEYELSVQIENMKDTATIRIRVVEK

9, 131, Ig, 131

PEIDLDVALRTSVIAKAGEDVQVLIPFKGRPPPTVTWRKDEKNLGSDARYSIENTDSSSLLTIPQVTRNDTGKYILTIENGVGEPKSSTVS

Score	Expect	Method	Identities	Positives	Gaps
56.2 bits(134)	4e-17	Compositional matrix adjust.	26/79(33%)	47/79(59%)	1/79(1%)

```
Query  1    PKIRLPRHLRQTYIRKVGEQLNLVVPFQGKPRPQVVWTKGGAPLDT-SRVHVRTSDFDTV  59
            P+I L   LR + I K GE + +++PF+G+P P V W K    L + +R  +  +D  ++
Sbjct  1    PEIDLDVALRTSVIAKAGEDVQVLIPFKGRPPPTVTWRKDEKNLGSDARYSIENTDSSSL  60

Query  60   FFVRQAARSDSGEYELSVQ  78
              + Q  R+D+G+Y L+++
Sbjct  61   LTIPQVTRNDTGKYILTIE  79
```

8, Ig, 6

PKIRLPRHLRQTYIRKVGEQLNLVVPFQGKPRPQVVWTKGGAPLDTSRVHVRTSDFDTVFFVRQAARSDSGEYELSVQIENMKDTATIRIRVVEK

10, 142, Ig, 134

PELDIDANFKQTHVVRAGASIRLFIAYQGRPTPTAVWSKPDSNLSLRADIHTTDSFSTLTVENCNRNDAGKYTLTVENNSGSKSIT

Score	Expect	Method	Identities	Positives	Gaps
55.1 bits(131)	1e-16	Compositional matrix adjust.	23/78(29%)	46/78(58%)	1/78(1%)

```
Query  1    PKIRLPRHLRQTYIRKVGEQLNLVVPFQGKPRPQVVWTKGGAPLDTSRVHVRTSDFDTVF  60
            P++ +  + +QT++ + G  + L + +QG+P P  VW+K  + L + R  + T+D  +
Sbjct  1    PELDIDANFKQTHVVRAGASIRLFIAYQGRPTPTAVWSKPDSNL-SLRADIHTTDSFSTL  59

Query  61   FVRQAARSDSGEYELSVQ  78
             V    R+D+G+Y L+V+
Sbjct  60   TVENCNRNDAGKYTLTVE  77
```

MyBP-C fast skeletal (human) and titin (human)

8, Ig, 6

PKIRLPRHLRQTYIRKVGEQLNLVVPFQGKPRPQVVWTKGGAPLDTSRVHVRTSDFDTVFFVRQAARSDSGEYELSVQIENMKDTATIRIRVVEK

11, 153, Ig, 137

PDLELADDLKKTVTIRAGASLRLMVSVSGRPPPVITWSKQGIDLASRAIIDTTESYSLLIVDKVNRYDAGKYTIEAENQSGKKSATVLVK

Score	Expect	Method	Identities	Positives	Gaps
52.8 bits(125)	9e-16	Compositional matrix adjust.	26/91(29%)	50/91(54%)	1/91(1%)

```
Query  1   PKIRLPRHLRQTYIRKVGEQLNLVVPFQGKPRPQVVWTKGGAPLDTSRVHVRTSDFDTVF  60
           P + L   L++T   + G  L L+V   G+P P + W+K G  L  SR  + T++   ++
Sbjct  1   PDLELADDLKKTVTIRAGASLRLMVSVSGRPPPVITWSKQGIDL-ASRAIIDTTESYSLL  59

Query  61  FVRQAARSDSGEYELSVQIENMKDTATIRIR  91
            V +  R D+G+Y +  + ++ K +AT+ ++
Sbjct  60  IVDKVNRYDAGKYTIEAENQSGKKSATVLVK  90
```

MyBP-C fast skeletal (human) and titin (human)

9, Fn, 3

PPINVMVKEVWGTNALVEWQAPKDDGNSEIMGYFVQKADKKTMEWFNVYERNRHTSCTVSDLIVGNEYYFRVYTENICGLSDSPGVSKNTARILKT

2, 63, Fn, 56

PPEKPEVSNVTKNTATVSWKRPVDDGGSEITGYHVERREKKSLRWVRAIKTPVSDLRCKVTGLQEGSTYEFRVSAENRAGIGPPSEASDSVLMKDA

Score	Expect	Method	Identities	Positives	Gaps
59.7 bits(143)	3e-18	Compositional matrix adjust.	31/81(38%)	41/81(50%)	1/81(1%)

```
Query  1    PPINVMVKEVWGTNALVEWQAPKDDGNSEIMGYFVQKADKKTMEWFNVYERN-RHTSCTV  59
            PP    V  V    A V W+ P DDG SEI GY V++ +KK++ W    +       C V
Sbjct  1    PPEKPEVSNVTKNTATVSWKRPVDDGGSEITGYHVERREKKSLRWVRAIKTPVSDLRCKV  60

Query  60   SDLIVGNEYYFRVYTENICGL  80
            + L  G+ Y FRV  EN  G+
Sbjct  61   TGLQEGSTYEFRVSAENRAGI  81
```

9, Fn, 3

PPINVMVKEVWGTNALVEWQAPKDDGNSEIMGYFVQKADKKTMEWFNVYERNRHTSCTVSDLIVGNEYYFRVYTENICGLSDSPGVSKNTARILKT

2, 64, Fn, 57

PPSNPHVTDTTKKSASLAWGKPHYDGGLEITGYVVEHQKVGDEAWIKDTTGTALRITQFVVPDLQTKEKYNFRISAINDAGVGEPAVIPDVEIVER

Score	Expect	Method	Identities	Positives	Gaps
37.0 bits(84)	1e-09	Compositional matrix adjust.	25/84(30%)	33/84(39%)	2/84(2%)

```
Query  1    PPINVMVKEVWGTNALVEWQAPKDDGNSEIMGYFVQKADKKTMEWFNVYERN--RHTSCT  58
            PP N  V +    +A + W  P  DG  EI GY V+        W         R T
Sbjct  1    PPSNPHVTDTTKKSASLAWGKPHYDGGLEITGYVVEHQKVGDEAWIKDTTGTALRITQFV  60

Query  59   VSDLIVGNEYYFRVYTENICGLSD  82
            V DL    +Y FR+   N  G+ +
Sbjct  61   VPDLQTKEKYNFRISAINDAGVGE  84
```

MyBP-C fast skeletal (human) and titin (human)

9, Fn, 3

PPINVMVKEVWGTNALVEWQAPKDDGNSEIMGYFVQKADKKTMEWFNVYERNRHTSCTVSDLIVGNEYYFRVYTENICGLSDSPGVSKNTARILKT

3, 74, Fn, 64

PPKNPEVTTITKDSMVVCWGHPDSDGGSEIINYIVERRDKAGQRWIKCNKKTLTDLRYKVSGLTEGHEYEFRIMAENAAGISAPSPTSPFYKACDT

Score	Expect	Method	Identities	Positives	Gaps
53.1 bits(126)	9e-16	Compositional matrix adjust.	29/82(35%)	40/82(48%)	1/82(1%)

```
Query  1   PPINVMVKEVWGTNALVEWQAPKDDGNSEIMGYFVQKADKKTMEWFNVYERN-RHTSCTV  59
           PP N  V  +   + +V W  P  DG SEI+ Y V++ DK    W    ++        V
Sbjct  1   PPKNPEVTTITKDSMVVCWGHPDSDGGSEIINYIVERRDKAGQRWIKCNKKTLTDLRYKV  60

Query  60  SDLIVGNEYYFRVYTENICGLS  81
           S L  G+EY FR+  EN  G+S
Sbjct  61  SGLTEGHEYEFRIMAENAAGIS  82
```

9, Fn, 3

PPINVMVKEVWGTNALVEWQAPKDDGNSEIMGYFVQKADKKTMEWFNVYERNRHTSCTVSDLIVGNEYYFRVYTENICGLSDSPGVSKNTARILKT

3, 75, Fn, 65

PPGNPRVLDTSRSSISIAWNKPIYDGGSEITGYMVEIALPEEDEWQIVTPPAGLKATSYTITGLTENQEYKIRIYAMNSEGLGEPALVPGTPKAEDR

Score	Expect	Method	Identities	Positives	Gaps
46.6 bits(109)	3e-13	Compositional matrix adjust.	33/97(34%)	44/97(45%)	5/97(5%)

```
Query  1   PPINVMVKEVWGTNALVEWQAPKDDGNSEIMGYFVQKADKKTMEWFNVYERN--RHTSCT  58
           PP N  V +   ++  + W  P  DG SEI GY V+ A  +  EW  V      + TS T
Sbjct  1   PPGNPRVLDTSRSSISIAWNKPIYDGGSEITGYMVEIALPEEDEWQIVTPPAGLKATSYT  60

Query  59  VSDLIVGNEYYFRVYTENICGLSDS---PGVSKNTAR  92
           ++ L    EY  R+Y  N  GL +    PG  K   R
Sbjct  61  ITGLTENQEYKIRIYAMNSEGLGEPALVPGTPKAEDR  97
```

MyBP-C fast skeletal (human) and titin (human)

9, Fn, 3

PPINVMVKEVWGTNALVEWQAPKDDGNSEIMGYFVQKADKKTMEWFNVYERNRHTSCTVSDLIVGNEYYFRVYTENICGLSDSPGVSKNTARILKT

4, 85, Fn, 72

APKAPEVTTVTKDSMIVVWERPASDGGSEILGYVLEKRDKEGIRWTRCHKRLIGELRLRVTGLIENHDYEFRVSAENAAGLSEPSPPSAYQKACDP

Score	Expect	Method	Identities	Positives	Gaps
55.5 bits(132)	1e-16	Compositional matrix adjust.	27/68(40%)	40/68(58%)	1/68(1%)

```
Query  16   LVEWQAPKDDGNSEIMGYFVQKADKKTMEWFNVYERN-RHTSCTVSDLIVGNEYYFRVYT  74
            +V W+ P  DG SEI+GY ++K DK+ + W   ++R        V+ LI  ++Y FRV
Sbjct  16   IVVWERPASDGGSEILGYVLEKRDKEGIRWTRCHKRLIGELRLRVTGLIENHDYEFRVSA  75

Query  75   ENICGLSD  82
            EN  GLS+
Sbjct  76   ENAAGLSE  83
```

9, Fn, 3

PPINVMVKEVWGTNALVEWQAPKDDGNSEIMGYFVQKADKKTMEWFNVYERNRHTSCTVSDLIVGNEYYFRVYTENICGLSDSPGVSKNTARILKT

4, 86, Fn, 73

PPNNPKVIDITRSSVFLSWSKPIYDGGCEIQGYIVEKCDVSVGEWTMCTPPTGINKTNIEVEKLLEKHEYNFRICAINKAGVGEHADVPGPIIVEEK

Score	Expect	Method	Identities	Positives	Gaps
45.1 bits(105)	1e-12	Compositional matrix adjust.	29/90(32%)	41/90(45%)	5/90(5%)

```
Query  1    PPINVMVKEVWGTNALVEWQAPKDDGNSEIMGYFVQKADKKTMEWFNVYERN--RHTSCT  58
            PP N  V ++  ++  + W  P  DG  EI GY V+K D    EW           T+
Sbjct  1    PPNNPKVIDITRSSVFLSWSKPIYDGGCEIQGYIVEKCDVSVGEWTMCTPPTGINKTNIE  60

Query  59   VSDLIVGNEYYFRVYTENICGL---SDSPG  85
            V  L+  +EY FR+   N  G+   +D PG
Sbjct  61   VEKLLEKHEYNFRICAINKAGVGEHADVPG  90
```

MyBP-C fast skeletal (human) and titin (human)

9, Fn, 3

PPINVMVKEVWGTNALVEWQAPKDDGNSEIMGYFVQKADKKTMEWFNVYERNRHTSCTVSDLIVGNEYYFRVYTENICGLSDSPGVSKNTARILKT

5, 96, Fn, 80

PPKSLEVTNIAKDSMTVCWNRPDSDGGSEIIGYIVEKRDRSGIRWIKCNKRRITDLRLRVTGLTEDHEYEFRVSAENAAGVGEPSPATVYYKACDP

Score	Expect	Method	Identities	Positives	Gaps
52.8 bits(125)	1e-15	Compositional matrix adjust.	28/83(34%)	41/83(49%)	1/83(1%)

```
Query  1   PPINVMVKEVWGTNALVEWQAPKDDGNSEIMGYFVQKADKKTMEWFNVYERN-RHTSCTV  59
           PP ++ V   +    +  V W  P  DG SEI+GY V+K D+  + W    +R        V
Sbjct  1   PPKSLEVTNIAKDSMTVCWNRPDSDGGSEIIGYIVEKRDRSGIRWIKCNKRRITDLRLRV  60

Query  60  SDLIVGNEYYFRVYTENICGLSD  82
           + L   +EY FRV  EN  G+ +
Sbjct  61  TGLTEDHEYEFRVSAENAAGVGE  83
```

9, Fn, 3

PPINVMVKEVWGTNALVEWQAPKDDGNSEIMGYFVQKADKKTMEWFNVYERNRHTSCTVSDLIVGNEYYFRVYTENICGLSDSPGVSKNTARILKT

5, 97, Fn, 81

PPTNAHIVDTTKNSITLAWGKPIYDGGSEILGYVVEICKADEEEWOIVTPOTGLRVTRFEISKLTEHOEYKIRVCALNKVGLGEATSVPGTVKPEDK

Score	Expect	Method	Identities	Positives	Gaps
43.1 bits(100)	7e-12	Compositional matrix adjust.	30/93(32%)	40/93(43%)	5/93(5%)

```
Query  1   PPINVMVKEVWGTNALVEWQAPKDDGNSEIMGYFVQKADKKTMEWFNVYERN--RHTSCT  58
           PP N  + +     +  + W  P  DG SEI+GY V+         EW  V  +   R T
Sbjct  1   PPTNAHIVDTTKNSITLAWGKPIYDGGSEILGYVVEICKADEEEWQIVTPQTGLRVTRFE  60

Query  59  VSDLIVGNEYYFRVYTENICGLSDS---PGVSK  88
           +S L    EY  RV   N  GL ++   PG  K
Sbjct  61  ISKLTEHQEYKIRVCALNKVGLGEATSVPGTVK  93
```

MyBP-C fast skeletal (human) and titin (human)

9, Fn, 3

PPINVMVKEVWGTNALVEWQAPKDDGNSEIMGYFVQKADKKTMEWFNVYERNRHTSCTVSDLIVGNEYYFRVYTENICGLSDSPGVSKNTARILKT

6, 107, Fn, 88

PPSTPEVSAITKDSMVVTWARPVDDGGTEIEGYILEKRDKEGVRWTKCNKKTLTDLRLRVTGLTEGHSYEFRVAAENAAGVGEPSEPSVFYRACDA

Score	Expect	Method	Identities	Positives	Gaps
51.6 bits(122)	3e-15	Compositional matrix adjust.	27/83(33%)	41/83(49%)	1/83(1%)

```
Query  1    PPINVMVKEVWGTNALVEWQAPKDDGNSEIMGYFVQKADKKTMEWFNVYERN-RHTSCTV  59
            PP    V  +   + +V W  P DDG +EI GY ++K DK+ + W    ++       V
Sbjct  1    PPSTPEVSAITKDSMVVTWARPVDDGGTEIEGYILEKRDKEGVRWTKCNKKTLTDLRLRV  60

Query  60   SDLIVGNEYYFRVYTENICGLSD  82
            + L  G+ Y FRV  EN  G+ +
Sbjct  61   TGLTEGHSYEFRVAAENAAGVGE  83
```

9, Fn, 3

PPINVMVKEVWGTNALVEWQAPKDDGNSEIMGYFVQKADKKTMEWFNVYERNRHTSCTVSDLIVGNEYYFRVYTENICGLSDSPGVSKNTARILKT

6, 108, Fn, 89

PPSNPKVTDTSRSSVSLAWSKPIYDGGAPVKGYVVEVKEAAADEWTTCTPPTGLQGKQFTVTKLKENTEYNFRICAINSEGVGEPATLPGSVVAQER

Score	Expect	Method	Identities	Positives	Gaps
34.3 bits(77)	2e-08	Compositional matrix adjust.	22/84(26%)	35/84(41%)	2/84(2%)

```
Query  1    PPINVMVKEVWGTNALVEWQAPKDDGNSEIMGYFVQKADKKTMEWFNVYERN--RHTSCT  58
            PP N  V +   ++  + W  P  DG + + GY V+  +    EW         +    T
Sbjct  1    PPSNPKVTDTSRSSVSLAWSKPIYDGGAPVKGYVVEVKEAAADEWTTCTPPTGLQGKQFT  60

Query  59   VSDLIVGNEYYFRVYTENICGLSD  82
            V+ L    EY FR+   N  G+ +
Sbjct  61   VTKLKENTEYNFRICAINSEGVGE  84
```

MyBP-C fast skeletal (human) and titin (human)

9, Fn, 3

PPINVMVKEVWGTNALVEWQAPKDDGNSEIMGYFVQKADKKTMEWFNVYERNRHTSCTVSDLIVGNEYYFRVYTENICGLSDSPGVSKNTARILKT

7, 118, Fn, 96

PPTSLEITSVTKESMTLCWSRPESDGGSEISGYIIERREKNSLRWVRVNKKPVYDLRVKSTGLREGCEYEYRVYAENAAGLSLPSETSPLIRAEDP

Score	Expect	Method	Identities	Positives	Gaps
54.7 bits(130)	2e-16	Compositional matrix adjust.	27/82(33%)	44/82(53%)	1/82(1%)

```
Query  1   PPINVMVKEVWGTNALVEWQAPKDDGNSEIMGYFVQKADKKTMEWFNVYERNRH-TSCTV  59
           PP ++ +  V   +  + W  P+ DG SEI GY +++ +K ++ W  V ++  +
Sbjct  1   PPTSLEITSVTKESMTLCWSRPESDGGSEISGYIIERREKNSLRWVRVNKKPVYDLRVKS  60

Query  60  SDLIVGNEYYFRVYTENICGLS  81
           + L  G EY +RVY EN  GLS
Sbjct  61  TGLREGCEYEYRVYAENAAGLS  82
```

9, Fn, 3

PPINVMVKEVWGTNALVEWQAPKDDGNSEIMGYFVQKADKKTMEWFNVYERNRHTSCTVSDLIVGNEYYFRVYTENICGLSDSPGVSKNTARILKT

7, 119, Fn, 97

PPSKPKIVDSGKTTITIAWVKPLFDGGAPITGYTVEYKKSDDTDWKTSIQSLRGTEYTISGLTTGAEYVFRVKSVNKVGASDPSDSSDPQIAKER

Score	Expect	Method	Identities	Positives	Gaps
48.9 bits(115)	4e-14	Compositional matrix adjust.	27/82(33%)	36/82(43%)	0/82(0%)

```
Query  1   PPINVMVKEVWGTNALVEWQAPKDDGNSEIMGYFVQKADKKTMEWFNVYERNRHTSCTVS  60
           PP    + +    T   + W  P  DG + I GY V+        +W    +  R T  T+S
Sbjct  1   PPSKPKIVDSGKTTITIAWVKPLFDGGAPITGYTVEYKKSDDTDWKTSIQSLRGTEYTIS  60

Query  61  DLIVGNEYYFRVYTENICGLSD  82
            L  G EY FRV + N  G SD
Sbjct  61  GLTTGAEYVFRVKSVNKVGASD  82
```

MyBP-C fast skeletal (human) and titin (human)

9, Fn, 3

PPINVMVKEVWGTNALVEWQAPKDDGNSEIMGYFVQKADKKTMEWFNVYERNRHTSCTVSDLIVGNEYYFRVYTENICGLSDSPGVSKNTARILKT

8, 129, Fn, 104

PPGIPEVTKITKNSMTVVWSRPIADGGSDISGYFLEKRDKKSLGWFKVLKETIRDTRQKVTGLTENSDYQYRVCAVNAAGQGPFSEPSEFYKAADP

Score	Expect	Method	Identities	Positives	Gaps
52.8 bits(125)	1e-15	Compositional matrix adjust.	29/80(36%)	42/80(52%)	1/80(1%)

```
Query  1   PPINVMVKEVWGTNALVEWQAPKDDGNSEIMGYFVQKADKKTMEWFNVYERN-RHTSCTV  59
           PP    V ++    +  V W  P  DG S+I GYF++K DKK++ WF V +    R T   V
Sbjct  1   PPGIPEVTKITKNSMTVVWSRPIADGGSDISGYFLEKRDKKSLGWFKVLKETIRDTRQKV  60

Query  60  SDLIVGNEYYFRVYTENICG  79
           + L   ++Y +RV   N  G
Sbjct  61  TGLTENSDYQYRVCAVNAAG  80
```

9, Fn, 3

PPINVMVKEVWGTNALVEWQAPKDDGNSEIMGYFVQKADKKTMEWFNVYERNRHTSCTVSDLIVGNEYYFRVYTENICGLSDSPGVSKNTARILKT

8, 130, Fn, 105

PPAKIRIADSTKSSITLGWSKPVYDGGSAVTGYVVEIRQGEEEEWTTVSTKGEVRTTEYVVSNLKPGVNYYFRVSAVNCAGQGEPIEMNEPVQAKDI

Score	Expect	Method	Identities	Positives	Gaps
44.7 bits(104)	1e-12	Compositional matrix adjust.	26/84(31%)	38/84(45%)	2/84(2%)

```
Query  1   PPINVMVKEVWGTNALVEWQAPKDDGNSEIMGYFVQKADKKTMEWFNVYERN--RHTSCT  58
           PP  + + +     ++  + W  P  DG S + GY V+    +  EW  V  +   R T
Sbjct  1   PPAKIRIADSTKSSITLGWSKPVYDGGSAVTGYVVEIRQGEEEEWTTVSTKGEVRTTEYV  60

Query  59  VSDLIVGNEYYFRVYTENICGLSD  82
           VS+L  G  YYFRV   N  G  +
Sbjct  61  VSNLKPGVNYYFRVSAVNCAGQGE  84
```

MyBP-C fast skeletal (human) and titin (human)

9, Fn, 3

PPINVMVKEVWGTNALVEWQAPKDDGNSEIMGYFVQKADKKTMEWFNVYERNRHTSCTVSDLIVGNEYYFRVYTENICGLSDSPGVSKNTARILKT

9, 140, Fn, 112

APGIPEPSNITGNSITLTWARPESDGGSEIQQYILERREKKSTRWVKVISKRPISETRFKVTGLTEGNEYEFHVMAENAAGVGPASGISRLIKCREP

```
Score           Expect  Method                        Identities    Positives     Gaps
57.0 bits(136)  3e-17   Compositional matrix adjust.  27/81(33%)    42/81(51%)    2/81(2%)

Query  10   VWGTNALVEWQAPKDDGNSEIMGYFVQKADKKTMEWFNVYERN--RHTSCTVSDLIVGNE  67
            + G +  + W  P+ DG SEI  Y +++ +KK+  W  V  +     T   V+ L  GNE
Sbjct  10   ITGNSITLTWARPESDGGSEIQQYILERREKKSTRWVKVISKRPISETRFKVTGLTEGNE  69

Query  68   YYFRVYTENICGLSDSPGVSK  88
            Y F V  EN  G+  + G+S+
Sbjct  70   YEFHVMAENAAGVGPASGISR  90
```

9, Fn, 3

PPINVMVKEVWGTNALVEWQAPKDDGNSEIMGYFVQKADKKTMEWFNVYERNRHTSCTVSDLIVGNEYYFRVYTENICGLSDSPGVSKNTARILKT

9, 141, Fn, 113

PPGPPTVVKVTDTSKTTVSLEWSKPVFDGGMEIIGYIIEMCKADLGDWHKVNAEACVKTRYTVTDLQAGEEYKFRVSAINGAGKGDSCEVTGTIKAVDR

```
Score           Expect  Method                        Identities    Positives     Gaps
52.8 bits(125)  1e-15   Compositional matrix adjust.  32/84(38%)    39/84(46%)    1/84(1%)

Query  1    PPINVMVKEVWGTNALVEWQAPKDDGNSEIMGYFVQKADKKTMEWFNV-YERNRHTSCTV  59
            PP  V V +   T   +EW  P  DG  EI+GY ++       +W  V  E    T  TV
Sbjct  4    PPTVVKVTDTSKTTVSLEWSKPVFDGGMEIIGYIIEMCKADLGDWHKVNAEACVKTRYTV  63

Query  60   SDLIVGNEYYFRVYTENICGLSDS  83
            +DL  G EY FRV   N  G  DS
Sbjct  64   TDLQAGEEYKFRVSAINGAGKGDS  87
```

MyBP-C fast skeletal (human) and titin (human)

9, Fn, 3

PPINVMVKEVWGTNALVEWQAPKDDGNSEIMGYFVQKADKKTMEWFNVYERNRHTSCTVSDLIVGNEYYFRVYTENICGLSDSPGVSKNTARILKT

10, 151, Fn, 120

PPGIPEEVGTGKEHIIIQWTKPESDGGNEISNYLVDKREKKSLRWTRVNKDYVVYDTRLKVTSLMEGCDYQFRVTAVNAAGNSEPSEASNFISCREP

Score	Expect	Method	Identities	Positives	Gaps
45.4 bits(106)	9e-13	Compositional matrix adjust.	25/73(34%)	39/73(53%)	10/73(13%)

```
Query  16   LVEWQAPKDDGNSEIMGYFVQKADKKTMEW------FNVYERNRHTSCTVSDLIVGNEYY  69
            +++W  P+ DG +EI  Y V K +KK++ W      + VY+    T   V+ L+ G +Y
Sbjct  16   IIQWTKPESDGGNEISNYLVDKREKKSLRWTRVNKDYVVYD----TRLKVTSLMEGCDYQ  71

Query  70   FRVYTENICGLSD  82
            FRV   N  G S+
Sbjct  72   FRVTAVNAAGNSE  84
```

9, Fn, 3

PPINVMVKEVWGTNALVEWQAPKDDGNSEIMGYFVQKADKKTMEWFNVYERNRHTSCTVSDLIVGNEYYFRVYTENICGLSDSPGVSKNTARILKT

10, 152, Fn, 121

PPSAPRVVDTTKHSISLAWTKPMYDGGTDIVGYVLEMQEKDTDQWYRVHTNATIRNTEFTVPDLKMGQKYSFRVAAVNVKGMSEYSESIAEIEPVER

Score	Expect	Method	Identities	Positives	Gaps
53.1 bits(126)	9e-16	Compositional matrix adjust.	28/84(33%)	46/84(54%)	2/84(2%)

```
Query  1    PPINVMVKEVWGTNALVEWQAPKDDGNSEIMGYFVQKADKKTMEWFNVYERN--RHTSCT  58
            PP     V +     +   + W   P  DG ++I+GY ++   +K T +W+ V+      R+T  T
Sbjct  1    PPSAPRVVDTTKHSISLAWTKPMYDGGTDIVGYVLEMQEKDTDQWYRVHTNATIRNTEFT  60

Query  59   VSDLIVGNEYYFRVYTENICGLSD  82
            V DL +G +Y FRV   N+ G+S+
Sbjct  61   VPDLKMGQKYSFRVAAVNVKGMSE  84
```

MyBP-C fast skeletal (human) and titin (human)

10, Ig, 7

PKFLTPLIDRVVVAGYSAALNCAVRGHPKPKVVWMKNKMEIREDPKFLITNYQGVLTLNIRRPSPFDAGTYTCRAVNELGEALAECKLEVRVPQ

3, 65, Ig, 114

PDFELDAELRRTLVVRAGLSIRIFVPIKGRPAPEVTWTKDNINLKNRANIENTESFTLLIIPECNRYDTGKFVMTIENPAGKKSGFVNVRVLD

Score	Expect	Method	Identities	Positives	Gaps
32.0 bits(71)	1e-07	Compositional matrix adjust.	19/71(27%)	32/71(45%)	2/71(2%)

```
Query  11   VVVAGYSAALNCAVRGHPKPKVVWMKNKMEIREDPKFLITNYQGVLTLNIRRPSPFDAGT  70
            VV AG S  +   ++G P P+V W K+ + ++       I N +    L I   + +D G
Sbjct  14   VVRAGLSIRIFVPIKGRPAPEVTWTKDNINLKNRAN--IENTESFTLLIIPECNRYDTGK  71

Query  71   YTCRAVNELGE  81
            +     N  G+
Sbjct  72   FVMTIENPAGK  82
```

10, Ig, 7

PKFLTPLIDRVVVAGYSAALNCAVRGHPKPKVVWMKNKMEIREDPKFLITNYQGVLTLNIRRPSPFDAGTYTCRAVNELGEALAECKLEVRVPQ

4, 76, Ig, 117

PEIELDADLRKVVTIRACCTLRLFVPIKGRPAPEVKWARDHGESLDKASIESTSSYTLLIVGNVNRFDSGKYILTVENSSGSKSAFVNVR

Score	Expect	Method	Identities	Positives	Gaps
28.1 bits(61)	3e-06	Compositional matrix adjust.	17/62(27%)	28/62(45%)	4/62(6%)

```
Query  24   VRGHPKPKVVWMKNKMEIREDPKFLIT-NYQGVLTLNIRRPSPFDAGTYTCRAVNELGEA  82
            ++G P P+V W ++  E  +      T +Y  ++  N+ R   FD+G Y     N  G
Sbjct  27   IKGRPAPEVKWARDHGESLDKASIESTSSYTLLIVGNVNR---FDSGKYILTVENSSGSK  83

Query  83   LA  84
             A
Sbjct  84   SA  85
```

MyBP-C fast skeletal (human) and titin (human)

10, Ig, 7

PKFLTPLIDRVVVAGYSAALNCAVRGHPKPKVVWMKNKMEIREDPKFLITNYQGVLTLNIRRPSPFDAGTYTCRAVNELGEALAECKLEVRVPQ

5, 87, Ig, 120

PDIDLDLELRKIINIRAGGSLRLFVPIKGRPTPEVKWGKVDGEIRDAAIIDVTSSFTSLVLDNVNRYDSGKYTLTLENSSGTKSAFVT

Score	Expect	Method	Identities	Positives	Gaps
35.4 bits(80)	5e-09	Compositional matrix adjust.	25/72(35%)	34/72(47%)	4/72(5%)

```
Query  14  AGYSAALNCAVRGHPKPKVVWMKNKMEIREDPKFLITNYQGVLTL-NIRRPSPFDAGTYT  72
            AG S  L   ++G P P+V W K   EIR+     +T+    L L N+ R   +D+G YT
Sbjct  17  AGGSLRLFVPIKGRPTPEVKWGKVDGEIRDAAIIDVTSSFTSLVLDNVNR---YDSGKYT  73

Query  73  CRAVNELGEALA  84
                N  G   A
Sbjct  74  LTLENSSGTKSA  85
```

10, Ig, 7

PKFLTPLIDRVVVAGYSAALNCAVRGHPKPKVVWMKNKMEIREDPKFLITNYQGVLTLNIRRPSPFDAGTYTCRAVNELGEALAECKLEVRVPQ

6, 98, Ig, 123

PELDLDSELRKGIVVRAGGSARIHIPFKGRPTPEITWSREEGEFTDKVQIEKGVNYTQLSIDNCDRNDAGKYILKLENSSGSKSAFVTVK

Score	Expect	Method	Identities	Positives	Gaps
33.5 bits(75)	3e-08	Compositional matrix adjust.	22/74(30%)	32/74(43%)	2/74(2%)

```
Query  11  VVVAGYSAALNCAVRGHPKPKVVWMKNKMEIREDPKFLITNYQGVLTLNIRRPSPFDAGT  70
            VV AG SA ++   +G P P++ W + + E  +  K  I        L+I      DAG
Sbjct  14  VVRAGGSARIHIPFKGRPTPEITWSREEGEFTD--KVQIEKGVNYTQLSIDNCDRNDAGK  71

Query  71  YTCRAVNELGEALA  84
            Y  +  N  G   A
Sbjct  72  YILKLENSSGSKSA  85
```

MyBP-C fast skeletal (human) and titin (human)

10, Ig, 7

PKFLTPLIDRVVVAGYSAALNCAVRGHPKPKVVWMKNKMEIREDPKFLITNYQGVLTLNIRRPSPFDAGTYTCRAVNELGEALAECKLEVRVPQ

7, 109, Ig, 126

PEIELDADLRKVVVLRASATLRLFVTIKGRPEPEVKWEKAEGILTDRAQIEVTSSFTMLVIDNVTRFDSGRYNLTLENNSGSKTAFVNVR

Score	Expect	Method	Identities	Positives	Gaps
30.8 bits(68)	3e-07	Compositional matrix adjust.	23/78(29%)	38/78(48%)	6/78(7%)

```
Query  10   RVVVAGYSAALN--CAVRGHPKPKVVWMKNKMEIREDPKFLITNYQGVLTL-NIRRPSPF  66
            +VVV   SA L     ++G P+P+V W K +  + +  +  +T+   +L + N+ R   F
Sbjct  11   KVVVLRASATLRLFVTIKGRPEPEVKWEKAEGILTDRAQIEVTSSFTMLVIDNVTR---F  67

Query  67   DAGTYTCRAVNELGEALA  84
            D+G Y     N  G   A
Sbjct  68   DSGRYNLTLENNSGSKTA  85
```

10, Ig, 7

PKFLTPLIDRVVVAGYSAALNCAVRGHPKPKVVWMKNKMEIREDPKFLITNYQGVLTLNIRRPSPFDAGTYTCRAVNELGEALAECKLEVRVPQ

8, 120, Ig, 129

PQIAKEREEEPLFDIDSEMRKTLIVKAGASFTMTVPFRGRPVPNVLWSKPDTDLRTRAYVDTTDSRTSLTIENANRNDSGKYTLTIQNVLSAASLT

Score	Expect	Method	Identities	Positives	Gaps
35.4 bits(80)	7e-09	Compositional matrix adjust.	23/72(32%)	32/72(44%)	2/72(2%)

```
Query  11   VVVAGYSAALNCAVRGHPKPKVVWMKNKMEIREDPKFLITNYQGVLTLNIRRPSPFDAGT  70
            +V AG S  +    RG P P V+W K   ++R       T+ +  LT  I   +  D+G
Sbjct  24   IVKAGASFTMTVPFRGRPVPNVLWSKPDTDLRTRAYVDTTDSRTSLT--IENANRNDSGK  81

Query  71   YTCRAVNELGEA  82
            YT    N L  A
Sbjct  82   YTLTIQNVLSAA  93
```

MyBP-C fast skeletal (human) and titin (human)

10, Ig, 7

PKFLTPLIDRVVVAGYSAALNCAVRGHPKPKVVWMKNKMEIREDPKFLITNYQGVLTLNIRRPSPFDAGTYTCRAVNELGEALAECKLEVRVPQ

8, 130, Fn, 105

PPAKIRIADSTKSSITLGWSKPVYDGGSAVTGYVVEIRQGEEEEWTTVSTKGEVRTTEYVVSNLKPGVNYYFRVSAVNCAGQGEPIEMNEPVQAKDI

Score	Expect	Method	Identities	Positives	Gaps
19.6 bits(39)	0.004	Compositional matrix adjust.	6/11(55%)	9/11(81%)	0/11(0%)

```
Query  18  AALNCAVRGHP  28
            +A+NCA +G P
Sbjct  75  SAVNCAGQGEP  85
```

10, Ig, 7

PKFLTPLIDRVVVAGYSAALNCAVRGHPKPKVVWMKNKMEIREDPKFLITNYQGVLTLNIRRPSPFDAGTYTCRAVNELGEALAECKLEVRVPQ

9, 131, Ig, 131

PEIDLDVALRTSVIAKAGEDVQVLIPFKGRPPPTVTWRKDEKNLGSDARYSIENTDSSSLLTIPQVTRNDTGKYILTIENGVGEPKSSTVS

Score	Expect	Method	Identities	Positives	Gaps
35.4 bits(80)	5e-09	Compositional matrix adjust.	19/68(28%)	29/68(42%)	0/68(0%)

```
Query  14  AGYSAALNCAVRGHPKPKVVWMKNKMEIREDPKFLITNYQGVLTLNIRRPSPFDAGTYTC  73
            AG    +    +G P P V W K++  +  D ++ I N      L I + +  D G Y
Sbjct  17  AGEDVQVLIPFKGRPPPTVTWRKDEKNLGSDARYSIENTDSSSLLTIPQVTRNDTGKYIL  76

Query  74  RAVNELGE  81
               N +GE
Sbjct  77  TIENGVGE  84
```

MyBP-C fast skeletal (human) and titin (human)

10, Ig, 7

PKFLTPLIDRVVVAGYSAALNCAVRGHPKPKVVWMKNKMEIREDPKFLITNYQGVLTLNIRRPSPFDAGTYTCRAVNELGEALAECKLEVRVPQ

10, 142, Ig, 134

PELDIDANFKQTHVVRAGASIRLFIAYQGRPTPTAVWSKPDSNLSLRADIHTTDSFSTLTVENCNRNDAGKYTLTVENNSGSKSIT

Score	Expect	Method	Identities	Positives	Gaps
34.7 bits(78)	9e-09	Compositional matrix adjust.	25/74(34%)	31/74(41%)	6/74(8%)

```
Query  10  RVVVAGYSAALNCAVRGHPKPKVVWMK--NKMEIREDPKFLITNYQGVLTLNIRRPSPFD  67
            VV AG S  L  A +G P P  VW K  + + +R D    I       TL +    + D
Sbjct  13  HVVRAGASIRLFIAYQGRPTPTAVWSKPDSNLSLRAD----IHTTDSFSTLTVENCNRND  68

Query  68  AGTYTCRAVNELGE  81
           AG YT     N  G
Sbjct  69  AGKYTLTVENNSGS  82
```

10, Ig, 7

PKFLTPLIDRVVVAGYSAALNCAVRGHPKPKVVWMKNKMEIREDPKFLITNYQGVLTLNIRRPSPFDAGTYTCRAVNELGEALAECKLEVRVPQ

11, 153, Ig, 137

PDLELADDLKKTVTIRAGASLRLMVSVSGRPPPVITWSKQGIDLASRAIIDTTESYSLLIVDKVNRYDAGKYTIEAENQSGKKSATVLVK

Score	Expect	Method	Identities	Positives	Gaps
37.7 bits(86)	7e-10	Compositional matrix adjust.	21/71(30%)	35/71(49%)	2/71(2%)

```
Query  14  AGYSAALNCAVRGHPKPKVVWMKNKMEIREDPKFLITNYQGVLTLNIRRPSPFDAGTYTC  73
           AG S  L  +V G P P + W K  +++    + +I   +    L + + + +DAG YT
Sbjct  17  AGASLRLMVSVSGRPPPVITWSKQGIDLAS--RAIIDTTESYSLLIVDKVNRYDAGKYTI  74

Query  74  RAVNELGEALA  84
            A N+ G+  A
Sbjct  75  EAENQSGKKSA  85
```
