# Supplementary material for: Formation of an Amyloid-like Structure During In Vitro Interaction of Titin and Myosin-Binding Protein C
Source: Int J Mol Sci. 2025 Jul 18;26(14):6910. doi: 10.3390/ijms26146910 (PMC12294915; doi:10.3390/ijms26146910)
Supplement: Supplementary file 1 [file ijms-26-06910-s001.zip › Supplementary titles.pdf]

Figure S1: Isolation of MyBP-C from rabbit skeletal muscle;
Figure S2: Isolation of titin from rabbit skeletal muscle;
Figure S3: Electron Microscopy of Titin–MyBPC Aggregates;
Figure S4: Atomic Force Microscopy of Titin–MyBPC Aggregates;
Figure S5: Atomic force microscopy of titin–MyBPC aggregates (analysis of images);
Figure S6: Amino acid sequence identity;
File S1: Electron microscopy of titin–MyBPC aggregates (analysis of images);
File S2: Secondary structure percentages from FTIR analysis;
File S3: Calculation of Amyloidogenic Regions;
File S4: Atomic force microscopy of titin–MyBPC aggregates (Statistical quantities).
